# Multiple TonB-dependent transport systems in *Helicobacter pylori*

**DOI:** 10.1128/iai.00018-26

**Published:** 2026-06-04

**Authors:** John T. Loh, Chiamaka D. Okoye, Dillon E. Kunkle, W. Hayes McDonald, Mark S. McClain, Eric P. Skaar, Timothy L. Cover

**Affiliations:** 1 Department of Medicine, Vanderbilt University School of Medicine 12327 , Nashville, Tennessee, USA; 2 Department of Pathology, Microbiology and Immunology, Vanderbilt University Medical Center 204907, Nashville, Tennessee, USA; 3 Mass Spectrometry Research Center, Vanderbilt University 12328, Nashville, Tennessee, USA; 4 Department of Biochemistry, Vanderbilt University 215875, Nashville, Tennessee, USA; 5 Vanderbilt Institute for Infection, Immunology, and Inflammation, Vanderbilt University Medical Center 12328, Nashville, Tennessee, USA; 6 Veterans Affairs Tennessee Valley Healthcare System, Nashville, Tennessee, USA; Stanford University School of Medicine, Stanford, California, USA

**Keywords:** TonB, TonB-dependent transporter, iron, nickel, metals, essential genes, protein complex

## Abstract

TonB-dependent transport systems (consisting of TonB-dependent transporters, TonB, ExbB, and ExbD) facilitate uptake of nutrients across the bacterial outer membrane. Tol-Pal systems contain components related to those of TonB systems. In this study, we investigated TonB-dependent transport systems in *Helicobacter pylori. H. pylori* genomes contain three *tonB* paralogs (designated *tonB1*, *tonB2*, and *tonB3/tolA*), three *exbB* paralogs, three *exbD* paralogs, six genes encoding putative TonB-dependent transporters, *tolB*, and *pal*. By using immunopurification and mass spectrometry methods, we show that TonB2, ExbB2, and ExbD2 co-purify with each other, consistent with the corresponding genes being in the same predicted operon. The *tonB1* gene is not linked to *exbB* or *exbD* genes, but we show that ExbB3 and ExbD3 co-purify with TonB1. The *tonB3/tolA* gene is in a predicted operon containing *exbB1/tolQ*, *exbD1/tolR*, *tolB*, and *pal*. We show that TonB3/TolA co-purifies with ExbB1/TolQ and ExbD1/TolR, suggesting that TonB3/TolA is a component of the Tol-Pal system. We show that *H. pylori tonB1* is essential for *H. pylori* growth, *tonB2* mutants require supplemental iron for growth, and *tonB3*/*tolA* is non-essential for growth *in vitro*. ICP-MS experiments showed that a *tonB2* mutant contains reduced levels of nickel compared to a wild-type strain, and correspondingly, the *tonB2* mutant exhibits reduced urease activity. These results indicate that the TonB2 system has an important role in *H. pylori* acquisition of nickel and iron. We propose that the TonB1 system has a key role in the acquisition of multiple nutrients required for *H. pylori* viability.

## INTRODUCTION

*Helicobacter pylori* is a Gram-negative bacterium that persistently colonizes the human gastric mucosa (1–3). *H. pylori* colonization results in gastritis and is associated with an increased risk of gastric cancer and peptic ulceration (1, 3). Within the gastric mucus layer, *H. pylori* likely encounters variations in environmental conditions, including pH and nutrient availability. Persistent colonization of the stomach is dependent on the ability of *H. pylori* to respond and adapt to these dynamic conditions (4).

Metal ions are a key class of nutrients that are incorporated into numerous metalloproteins and essential enzymes (5–7). About one-third of the proteins in all organisms require a metal ion to function. For example, iron (Fe) is a vital metal involved in electron transport and DNA replication (8), and it serves as a cofactor for iron-sulfur proteins (9). Iron has also been implicated in the pathogenesis of gastric diseases linked to *H. pylori*. Studies in animal models show that dietary iron deficiency can enhance the risk of gastric dysplasia and adenocarcinoma (10, 11)

Nickel is a trace metal that is essential for *H. pylori* colonization of the human gastric mucosa (12–15). Nickel is incorporated into *H. pylori* urease, a nickel-dependent metalloenzyme that hydrolyzes urea into ammonia, which buffers the acidic pH of the stomach, enabling acid resistance (16–18). Additionally, nickel is essential for the activity of hydrogenase, an enzyme that utilizes molecular hydrogen as an energy source within the gastric niche (19–21). These nickel-dependent enzymes are important for *H. pylori* colonization of the stomach.

*H. pylori* acquires metals in multiple ways, including the use of one or more TonB-dependent transport systems (22–24). TonB-dependent systems are comprised of three inner membrane proteins (TonB, ExbB, and ExbD) that form a complex to harvest the proton-motive force of the inner membrane and transduce that energy to the outer membrane (25–28). TonB, the energy transducer, spans the periplasmic space and interacts with TonB-dependent transporters (TBDTs) embedded in the outer membrane. TonB induces conformational changes in TBDTs that facilitate the passage of the target metal ion from the extracellular environment into the periplasm (25–27, 29, 30). Tol-Pal systems, which have an important role in maintaining outer membrane stability, contain components related to those of TonB systems (28, 31). Both TonB and Tol-Pal systems transduce energy from the proton-motive force of the cytoplasmic membrane to the outer membrane (28, 32).

TonB-dependent transport systems are present in many Gram-negative bacterial species. Some species contain a single TonB-dependent transport system, and others contain multiple TonB-dependent transport systems (33–35). Annotations of several *H. pylori* genomes (e.g., strains B8, G27, and J166) (36–38) designate three paralogs of *tonB* (*tonB1*, *tonB2*, *tonB3*). An initial annotation of the *H. pylori* reference genome (strain 26695) (23) identified only a single *tonB* gene (HP1341, corresponding to *tonB2*), but subsequently, additional *tonB* genes have been designated, including HP0582 (*tonB1*) and HP1128/HP1127 (two gene fragments corresponding to *tonB3*) (36, 39). The *tonB3* gene might encode either a TonB protein (36) or the TolA component of a Tol-Pal system (28, 40–42). In the current study, we designate HP1128/1127 as *tonB3/tolA. H. pylori* genomes also contain three *exbB* paralogs, three *exbD* paralogs, and six genes predicted to encode TonB-dependent transporters (TBDTs), which have been named *fecA-* or *frpB*-like genes (23, 24, 36).

The genes encoding components of TonB-dependent transport systems are annotated inconsistently among various *H. pylori* strains. For example, the three *tonB* paralogs in *H. pylori* strain B8 are annotated as *tonB1*, *tonB3*, and *tonB5* (36). Moreover, *tonB2* in reference strain 26695 is annotated as *tonB1* in strain B8, while *tonB1* in strain 26695 (23) is annotated as *tonB5* in strain B8 (36). In the current manuscript, we use the gene designations shown in Table 1. Specifically, we designate HP1341 as *tonB2* and HP0582 as *tonB1*, and we designate HP1128/HP1127 as *tonB3/tolA*, consistent with the nomenclature in several previous publications (24, 39, 43).

**TABLE 1:** Gene names and corresponding gene numbers in *H. pylori* strain 26695 and *H. pylori* B8

Gene	26695 number	B8 gene number^*a*^
TonB1	HP0582	HPB8_779
TonB2	HP1341	HPB8_138
TonB3/TolA^*b*^^,*c*^	HP1127/HP1128	HPB8_373
ExbB1/TolQ^*c*^^,*d*^	HP1130	HPB8_371
ExbB2	HP1339	HPB8_140
ExbB3	HP1445	HPB8_91
ExbD1/TolR^*c*^^,*e*^	HP1129	HPB8_372
ExbD2	HP1340	HPB8_139
ExbD3	HP1446	HPB8_90

^*a*^ B8 gene numbers are listed. *H. pylori* strain 7.13 (44) and *H. pylori* B8 (36) are derived from *H. pylori* B128 (45). Gene sequences from the *H. pylori* B8 genome were used to design plasmids to introduce mutations into the *H. pylori* 7.13 genome.

^*b*^ BLAST search yields either TonB or TolA.

^*c*^ Genome organization suggests protein to be located among genes with Tol-Pal function.

^*d*^ Annotated as *exbB* in *H. pylori* 26695 and *H. pylori* B8.

^*e*^ Annotated as *exbD* in *H. pylori* 26695 and *H. pylori* B8.

Thus far, there have been relatively few studies of TonB-dependent transport systems in *H. pylori*. It is unclear how the multiple paralogs of TonB, ExbB, ExbD, and TBDTs are physically or functionally related, and the functions of most of these proteins have not been investigated in detail. Previous studies have characterized *H. pylori tonB2* mutants, and *H. pylori* TonB2 is reported to have a role in nickel transport (22). One of the *H. pylori* TBDTs, designated FrpB4 (HP1512) in *H. pylori* strain 26695, has also been implicated in nickel transport (22, 43). The functions of the remaining TBDTs have been inferred based on sequence relatedness to TBDTs in *Escherichia coli*, coupled with studies of the regulatory influence of specific transcriptional regulators or metals on the expression of individual TBDTs. For instance, FecA1 and FecA2 (HP0686 and HP0807 in *H. pylori* strain 26695) are predicted to have roles in iron acquisition, based on the capacity of Ferric uptake regulator (Fur) and iron to modulate *fecA1* and *fecA2* transcription (46). Functional properties of *tonB1* mutants or *tonB3/tolA* mutants have not yet been reported.

In the current study, we analyzed physical interactions among *H. pylori* TonB, ExbB, ExbD, and TBDT proteins, and we analyzed properties of *tonB* mutant strains. We show that *H. pylori tonB1* is an essential gene, *tonB2* mutants require supplemental iron for growth, and *tonB3*/*tolA* is non-essential for growth *in vitro*. We show that the TonB2 system has an important role in *H. pylori* acquisition of nickel, and we propose that the TonB1 system has a key role in the acquisition of multiple nutrients required for *H. pylori* viability.

## RESULTS

### *H. pylori* genomes commonly contain three *tonB* paralogs

As a starting point for investigating *H. pylori* TonB systems, we analyzed the genomes of several well-characterized *H. pylori* strains. Three *tonB* paralogs (*tonB1*, *tonB2*, *tonB3/tolA*) are annotated in the genomes of *H. pylori* strains J166, G27, and B8 (36–38). In *H. pylori* strain B8, genes encoding these *tonB* paralogs are designated *HPB8_138*, *HPB8_373*, and *HPB8_779* (Table 1). The annotations of several other *H. pylori* genomes (26695, J99, and P12) designate fewer *tonB* paralogs (23, 24, 47). For example, only one *tonB* paralog (i.e., *tonB2, HP1341*, Table 1) was designated in the original annotation of the genome of reference strain 26695 (23). Our analyses suggest that the 26695 genome contains two additional *tonB* paralogs, designated *tonB1* (*HP0582*) and *tonB3/tolA* (*HP1128/HP1127*, Table 1). In the originally reported genome sequence *H. pylori* 26695 (24), a nucleotide insertion (c) at position 249 is predicted to cause the premature termination of the N-terminal domain of *tonB3/tolA* (*HP1128*).

**Fig 1:**
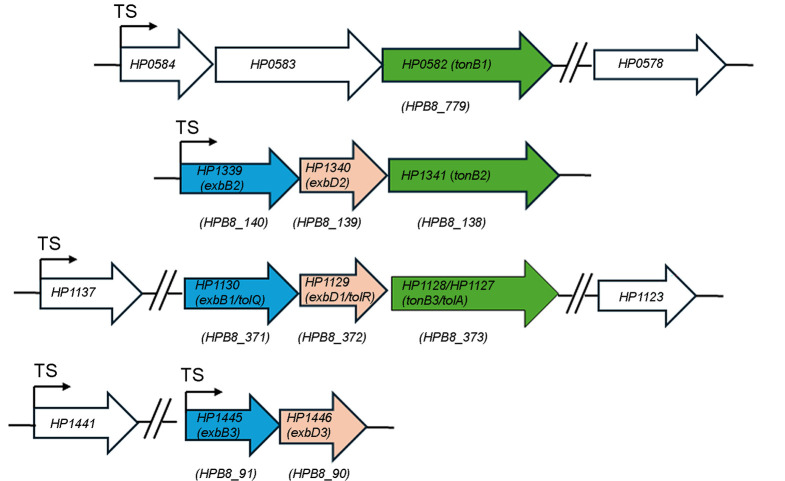
*H. pylori* genes annotated as *tonB*, *exbB,* or *exbD*. The figure shows relevant genes in reference strain *H. pylori* 26695 (23) (HP numbers and boxed colored arrows) and *H. pylori* B8 (36) (HPB8 numbers in parentheses). Experiments in this study were performed using *H. pylori* strain 7.13 (44), which is closely related to *H. pylori* strain B8. *H. pylori* genes *exbB2-exbD2-tonB2* are predicted to be transcribed from a single operon (second line of figure) (48). *H. pylori* gene *tonB1* (HP0582) is not linked to *exbB* or *exbD* genes (top line of figure) (48). *H. pylori* genes *exbB1/tolQ*, *exbD1/tolR*, and HP1128/1127 (*tonB3/tolA*) (third line of figure) are predicted to be transcribed in an operon (48) that contains genes encoding putative members of the Tol-Pal system (see Fig. S6 for Tol-Pal genome organization and Table 1 for gene designations). HP1128 and HP1127 in the genome of strain 26695 are gene fragments that each encode a portion of TonB3/TolA. Predicted operons (HP0584 to HP0578, HP1339 to HP1341, HP1137 to HP1123, and HP1441 to HP1446) are shown (48). TS indicates transcriptional start sites for each of the operons (48).

In strain 26695 (23, 48), the *tonB2* gene (*HP1341*) is part of a gene cluster that includes *exbB* and *exbD* paralogs (*exbB2* and *exbD2*) (Table 1). The *tonB1* gene (*HP0582*) is not linked to *exbB* or *exbD* paralogs (Fig. 1) (23). Similarly, *exbB3* and *exbD3* are not linked to *tonB* paralogs (Fig. 1) (23). The *tonB3/tolA* gene (*HP1128/HP1127*) is in a gene cluster that includes *exbB* and *exbD* paralogs, designated *exbB1/tolQ* and *exbD1/tolR* (Fig. 1) (23). This gene cluster also includes genes predicted to encode components of a Tol-Pal system. Therefore, HP1128/HP1127 might be a component of the Tol-Pal system, corresponding to TolA.

**Fig 2:**
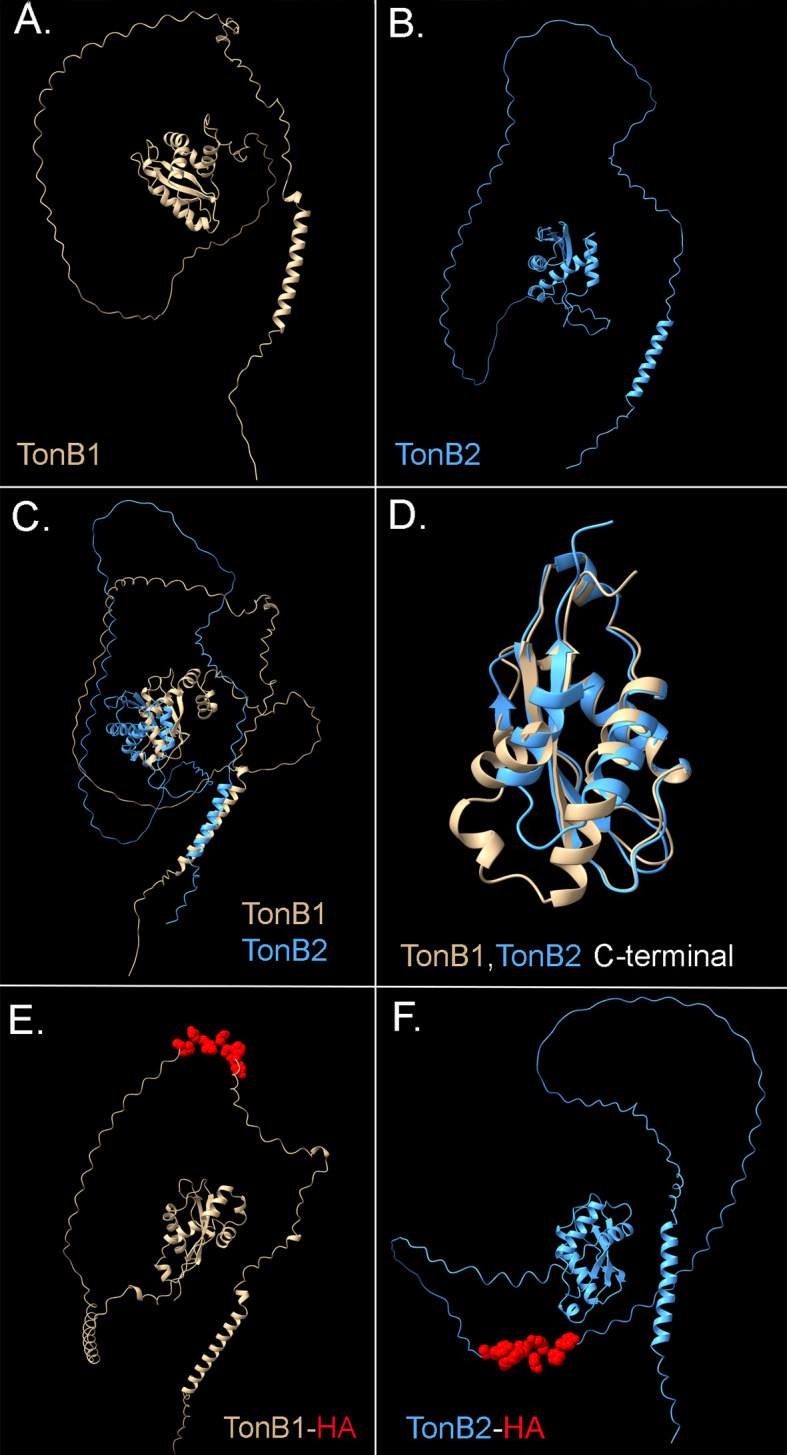
Predicted structures of *H. pylori* TonB1 and TonB2 from *H. pylori* strain 26695, based on AlphaFold 3 modeling. (**A and B**) TonB1 (HP0582) (**A**) and TonB2 (HP1341) (**B**) are predicted to have closely related structures. (**C**) Comparison of the AlphaFold 3-modeled structures of TonB1 and TonB2 structures (whole protein). RMSD (root mean square deviation) of atomic positions was calculated using UCSF ChimeraX 1.10.1 (50–52), and RMSD values are reported either across all atom pairs or pruned by ChimeraX to discard outlier atom pairs (cutoff > 2 Å). The RMSD across all 286 atom pairs is 26.66 Å, a high number due to predicted flexible regions or very low confidence regions throughout the protein (i.e., pLDDT [predicted local distance difference test] <50). (**D**) Comparison of AlphaFold 3 modeled C-terminal structures of TonB1 (i.e., aa 203–317) and TonB2 (i.e., aa 190–287). The C-terminal structure of both proteins is modeled by AlphaFold 3 with very high confidence (pLDDT > 90). RMSD across all 107 atom pairs is 3.14 Å, while the RMSD between 86 pruned atom pairs is 0.80 Å. A predicted structure of *H. pylori* TonB3/TolA is shown in Fig. S2A. (**E and F**) The locations of HA tags inserted into the proline-rich rod structure of TonB1 or TonB2 are shown in red in panels **E **and **F**. Strains harboring the HA-tagged TonB1 or TonB2 proteins were used in immunoprecipitation experiments.

TonB1 and TonB2 proteins have very little similarity in amino acid sequence (Fig. S1), but there is a high level of similarity between the predicted structures of the two proteins (Fig. 2A through C), including the C-terminal domain (Fig. 2D). Both TonB1 and TonB2 contain predicted structural domains characteristic of bacterial TonB proteins (49), including a hydrophobic N-terminal α-helical domain, a proline-rich flexible spacer domain composed of Pro-Lys repeats and glutamic acid residues, and a C-terminal domain (Fig. 2A and B; Fig. S1). The proline-rich spacer domain is predicted to span the periplasmic space, and the C-terminal domain is predicted to interact with outer membrane transporters (49). The predicted structure of TonB3/TolA exhibits similarities to structures of TonB1 and TonB2, but there are notable differences (discussed subsequently) (see Fig. S2).

To further study the functions of *H. pylori tonB* genes, we undertook experiments to generate *tonB1*, *tonB2*, and *tonB3/tolA* mutants, as described in the “Materials and Methods” section. For these mutagenesis experiments, we used *H. pylori* 7.13 as the parental strain (44). Strain 7.13 is closely related to *H. pylori* strain B8 (36), a strain in which three *tonB* paralogs are annotated in the genome. Both strains 7.13 and B8 are derived from strain B128, and the genetic organization of *tonB* paralogs and related genes is identical in whole genome sequences for both 7.13 and B8 strains (36, 44, 45, 53).

### TonB2 mutant requires iron for growth

**Fig 3:**
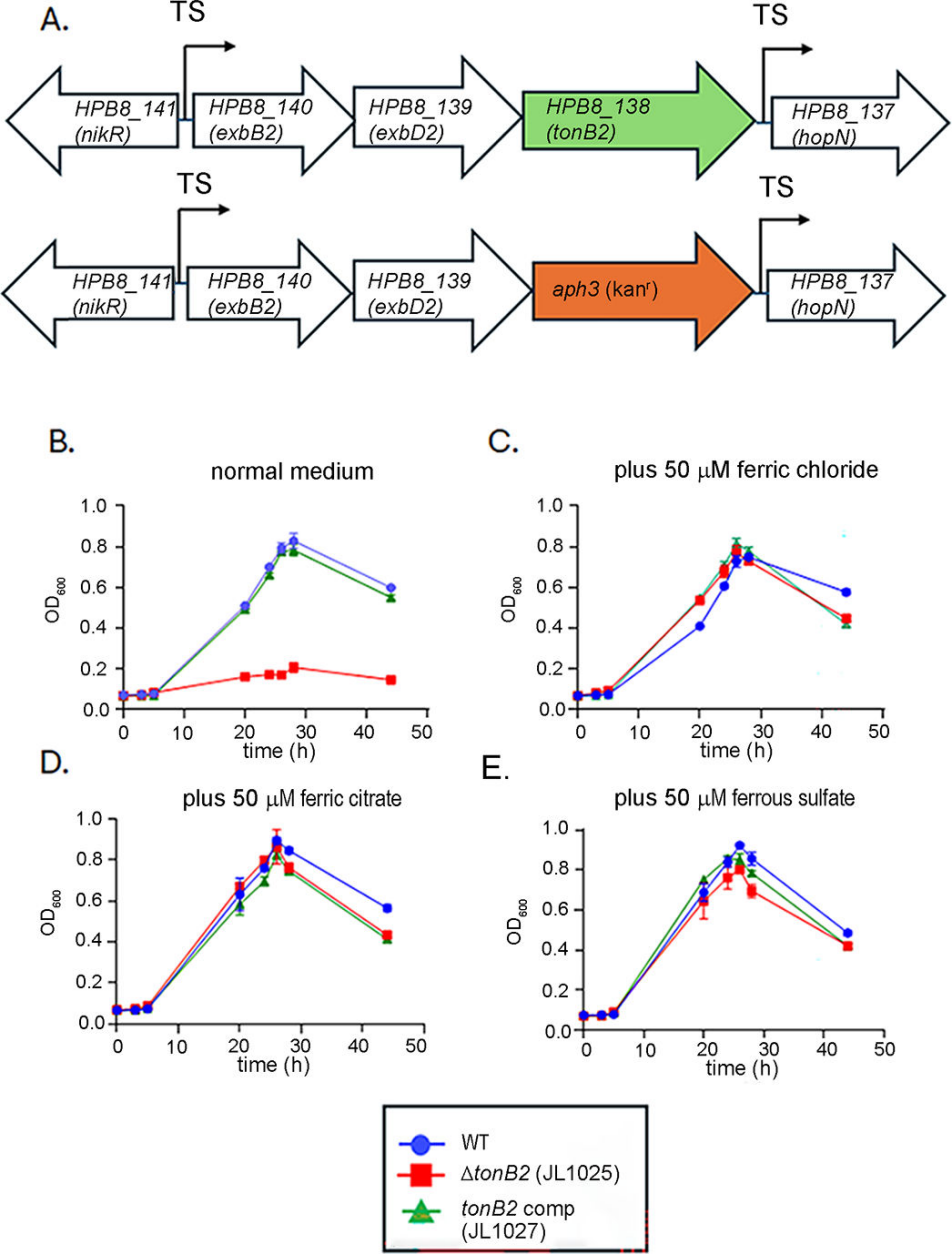
*H. pylori* TonB2 mutant requires supplemental iron for growth. (**A**) Method for generating a TonB2 mutant. The *tonB2* gene in *H. pylori* strain 7.13 (green) was deleted and replaced with an *aph3* gene, which confers kanamycin resistance (orange). Δ*tonB2* strains were generated in the presence of 1 mM FeCl_3_, as described in a previous study (22). (**B–E**) WT, *ΔtonB2* mutant (i.e., strain JL1025, Table 2), and complemented *tonB2* strains (strain JL1027, Table 2), grown on agar plates containing 1 mM FeCl_3_, were resuspended and cultured in fresh medium (OD_600_ ~ 0.1) containing no supplemental iron (**B**), 50 µM ferric chloride, ferric citrate, or ferrous sulfate (**C–E**). OD_600_ values were recorded at the indicated time points.

As a first step in generating a Δ*tonB2* mutant, we synthesized a plasmid designed to delete *tonB2* and replace it with an *aph3* cassette (Fig. 3A). Based on previous work (22), we used medium containing supplemental iron (along with kanamycin) and successfully selected for the Δ*tonB2::aph3* mutant, hereafter designated JL1025 (Table 2). In parallel, we attempted to generate a Δ*tonB2* mutant in the absence of supplemental iron, but we were unable to recover any Δ*tonB2* mutants. To further evaluate an apparent iron requirement of the Δ*tonB2* mutant, we cultured the WT and Δ*tonB2* strains individually on *Brucella-*FBS (BB-FBS) agar plates containing supplemental iron, and then, we inoculated the strains into BB-FBS medium (Materials and Methods) or BB-FBS medium supplemented with iron (50 µM). As shown in Fig. 3B, little to no growth of the Δ*tonB2* mutant JL1025 was observed in routine medium (without supplemental iron). To ensure that the phenotype observed with JL1025 was a result of the *tonB2* mutation, we used a two-step antibiotic counterselection protocol (described in the “Materials and Methods” section) for complementation. We first inserted an *aacC4-rpsL* cassette between the *neu* and *efp* genes of a streptomycin-resistant (*rpsL*-K43R) mutant of JL1025, generating strain JL1026. The *aacC4* gene confers resistance to gentamicin, and streptomycin susceptibility is mediated by the intact *rpsL* gene from *H. pylori* 26695. We next replaced the *aacC4-rpsL* cassette by introducing a copy of the intact *tonB2* gene into the intergenic region between *neu* and *efp* genes (54) of the Δ*tonB2* mutant JL1026, yielding strain JL1027 (Table 2). In contrast to the Δ*tonB2* mutant, strain JL1027 showed growth equivalent to that of the WT strain in routine medium (without supplemental iron) (Fig. 3), indicating complementation of the Δ*tonB2* phenotype in strain JL1025. The Δ*tonB2* mutant JL1025 grew in medium supplemented with 50 µM ferric chloride (Fig. 3C), ferric citrate (Fig. 3D), or ferrous sulfate (Fig. 3E). Growth of the Δ*tonB2* mutant under the iron-supplemented conditions was as robust as that of the WT strain and strain JL1027 (Table 2).

**TABLE 2:** List of strains and plasmids used in this study

Name	Relevant characteristics	Reference
Strains		
7.13	*H. pylori* wild type	(44)
JL1025	7.13 Δ*tonB2::aph3*, kan^R^	This study
JL1026	JL1025; *rpsL-K43R*, *neuB-efp::aacC4-rspL,* Gent*^R^*	This study
JL1027	JL1026, *neuB-efp::tonB2*, Sm^R^	This study
JL1028	JL1026, *neuB-efp::tonB2-HA,* Sm*^R^*	This study
JL1029	7.13, *rpsL*-K43R, *neuB-efp::aacC4-rpsL,* Gent*^R^*	This study
JL1030	JL1029, *neuB-efp::tonB1-HA,* Sm*^R^*	This study
JL1031	JL1030, Δ*tonB1::cat, neuB-efp::tonB1-HA, Sm^R^,* Cm*^R^*	This study
JL1032	7.13 Δ*tonB3/tolA::aph3*, kan^R^	This study
JL1033	JL1032*,* Δ*tonB3/tolA::aph3, neuB-efp::tonB3/TolA-HACAT, kan^R^,* Cm*^R^*	This study
Plasmids		
pTB2	pGEMT::*rpslK43R*; lysine at amino acid 43 of RpsL replaced with an arginine, Amp^R^	(55, 56)
p1341kan	pUC57, contains an *aph3* cassette inserted between nucleotides 500 bp upstream and downstream of the deleted *tonB2* gene (Genscript), Amp^R^, Kan^R^	This study
p1128/1127kan	pUC57, contains an *aph3* cassette inserted between nucleotides 500 bp upstream and downstream of the deleted *tonB3* gene (Genscript), Amp^R^, Kan^R^	This study
p582cat	pUC57, contains a *cat* cassette inserted between nucleotides 500 bp upstream and downstream of the deleted *tonB1* gene (Genscript), Amp^R^, Cm^R^	This study
ptonB2 HA	pUC57::*tonB2 HA*. HA Tag inserted following proline 134 of TonB2 (Genscript). tonB2 HA introduced into the *neuB-efp* locus, Amp^R^	This study
p177-aacC4-rpsL	pGEMT based. *aacC4-rpsL cassette* (71) inserted into the cloned *neuB-efp* locus. Amp^R^, Gent^R^.	This study
ptonB2	pUC57::*tonB2*. plasmid used to introduce the tonB2 gene into the *neuB-efp* locus, Amp^R^ (Genscript).	This study
ptonB1 HA	pUC57::*tonB1 HA*. HA Tag inserted following proline 134 of TonB2 (Genscript). *tonB1 HA* introduced into the *neuB-efp* locus, Amp^R^	This study
ptonB3 HA-CAT	pUC57::*tonB3 HA*. HA Tag inserted following Glutamate 81 of TonB3 (Genscript). *tonB3 HA* introduced into the *neuB-efp* locus, Amp^R^, Cm^R^	This study

### Role of TonB2 in nickel acquisition

**Fig 4:**
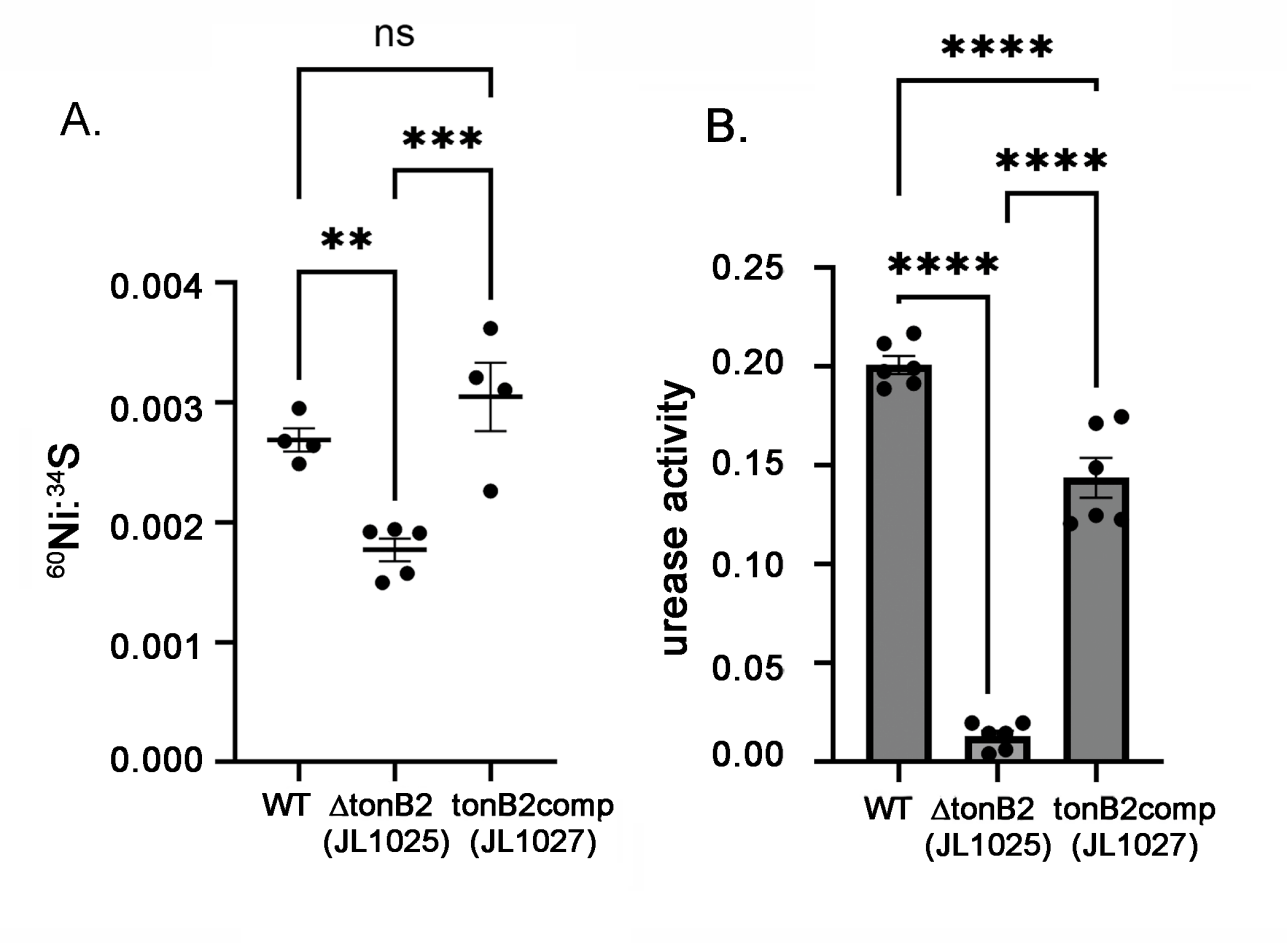
Reduced urease activity and nickel content of a Δ*tonB2* mutant strain. (**A**) Cultures were grown overnight in medium containing 25 µM FeCl_3_, washed, and then resuspended and cultured in medium containing 5 µM FeCl_3_ for 7 h. Cultures were fixed with acetone/EtOH and pelleted cells were used in ICP-MS analyses, as described in the “Materials and Methods” section. (**B**) *H. pylori* strains (WT, *ΔtonB2* [i.e., strain JL1025] and complemented *tonB2* mutant [strain JL1027]) were cultured overnight in *Brucella* broth (pH 7.0) supplemented with 25 µM FeCl_3_ and then passaged for 2 h in fresh *Brucella* broth (pH 5.3) supplemented with 5 µM FeCl_3_. Urease activity in cells was determined as described in the “Materials and Methods” section. One-way ANOVA (****P < 0.0001, ***P = 0.0009, **P = 0.0083. ns, *P*>0.05 ).

A previous study reported that an *H. pylori* Δ*tonB2* mutant exhibited a defect in nickel acquisition (22). To corroborate those results, we analyzed the JL1025 mutant (Δ*tonB2::aph3*) and complemented mutant strain JL1027 (*7.13* Δ*tonB2::aph3*, *neuB-efp::tonB2*) using ICP-MS techniques. As shown in Fig. 4A, the Δ*tonB2* mutant JL1025 demonstrated reduced concentrations of nickel compared to the WT strain. The nickel content was restored in the complemented *tonB2* mutant JL1027.

Nickel serves as a critical cofactor for urease activity. Given the observed reduction in intracellular nickel levels in the Δ*tonB2* mutant, we analyzed and compared urease activity in the Δ*tonB2* mutant compared to wild-type and complemented mutant strains. Consistent with diminished nickel availability, urease activity was significantly reduced in the Δ*tonB2* mutant strain JL1025 (Fig. 4B). Notably, complementation with a wild-type *tonB2* allele in JL1027 restored urease activity to levels comparable with those of the wild-type strain, indicating a role for TonB2 in nickel acquisition essential for urease function. Reduced urease activity has previously been reported in a mutant harboring a deletion of the *tonB2-exbB2-exbD2* operon (22). Thus, the properties of the Δ*tonB2* mutant JL1025 match previously reported results.

### TonB1 is essential for *H. pylori* growth

**Fig 5:**
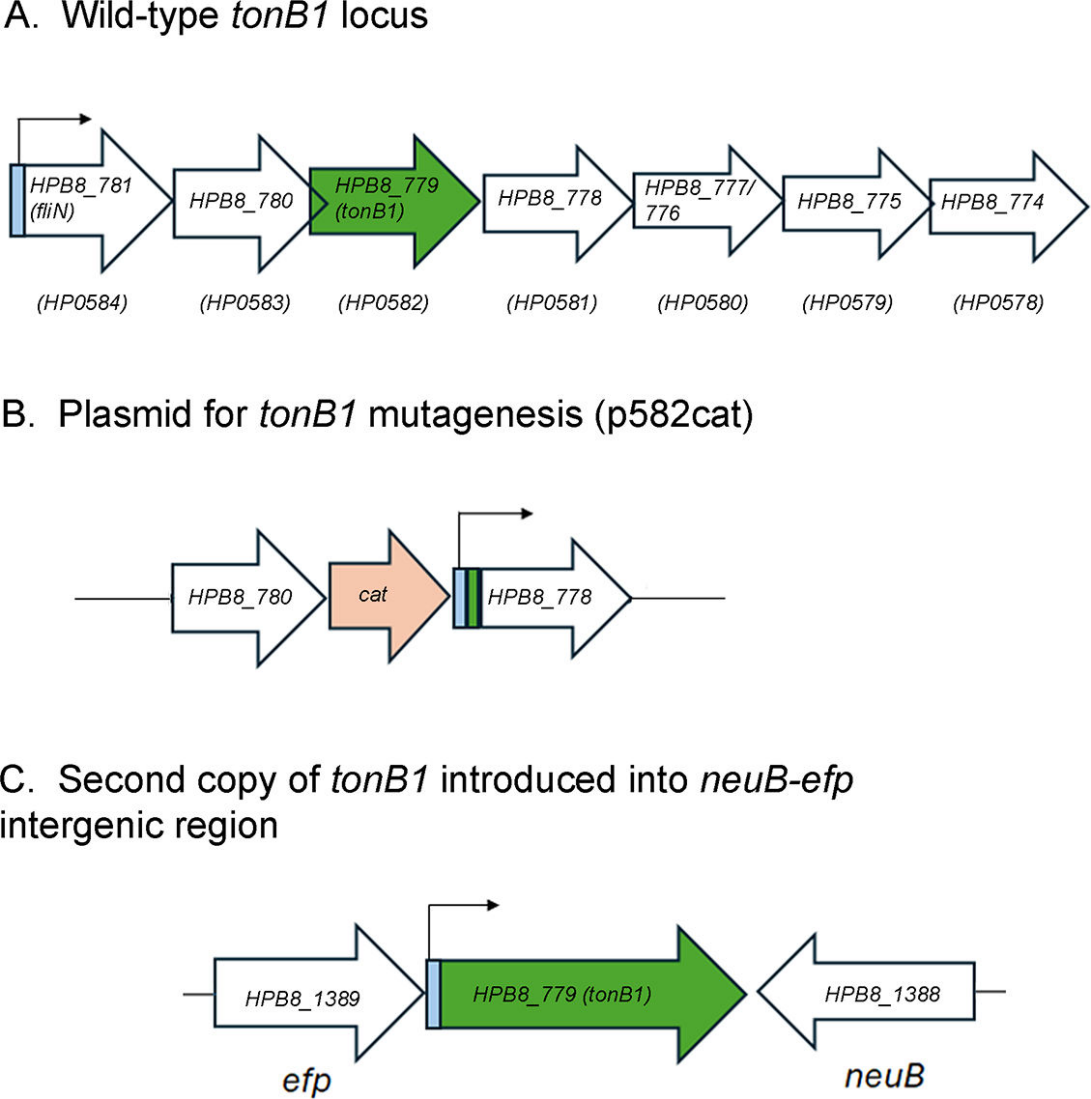
Schematic illustrating method used to mutate the endogenous *tonB1* gene. (**A**) Analysis of the *H. pylori* B8 genome (36) indicates that TonB1 is the third gene in a predicted operon extending from HP0584 (HPB8_781) to HP0578 (HPB8_774). (**B**) The plasmid in panel **B **was designed to replace HPB8_779 (*tonB1*) with a chloramphenicol acetyltransferase (*cat*) antibiotic selection marker (conferring chloramphenicol resistance). Sequences inserted upstream of the HPB8_778 gene contained the HPB8_781 promoter and 3′ region of *tonB1* (containing the Shine-Dalgarno RBS binding site for HPB8_778). Analysis of 30 transformants (from multiple independent transformations) revealed that all the chloramphenicol-resistant isolates resulted from single-crossover events that retained an intact *tonB1* gene. (**C**) To confirm the essentiality of the *tonB1* gene, a second copy of *tonB1* was introduced into a heterologous locus (*neuB-efp* intergenic region, corresponding to HPB8_1388_HPB8_1389). The resulting strain, containing two copies of *tonB1*, was transformed with the plasmid shown in panel **B**. 100% of chloramphenicol-resistant strains harbored a replacement of the endogenous *tonB1 gene* with the chloramphenicol cassette. Gene numbers are shown for both *H. pylori* B8 and *H. pylori* 26695 (in parentheses, panel **A**).

Figure 5A illustrates that *tonB1* in the strain chosen for mutagenesis (strain 7.13) is localized within a predicted operon containing seven genes. The genes in this predicted operon, based on the *H. pylori* B8 genome nomenclature (36), include HPB8_781 (encoding flagellar motor switch protein FliN), HPB8_778 (*pyrC*, conferring dihydroorotase activity in pyrimidine biosynthesis), HPB8_776 (encoding predicted sialidase), and three genes encoding hypothetical proteins (HPB8_780, HPB8_777, HPB8_775). As a first step in generating a *tonB1* mutant, we synthesized a plasmid (Fig. 5B) designed to replace the *tonB1* gene (designated *HP0582* in *H. pylori* 26695 and *HPB8_779* in strain B8) (Table 1) with a chloramphenicol acetyltransferase (*cat*) cassette. As *tonB1* is located in an operon, we were concerned that the disruption of *tonB1* may impact the expression of downstream genes in the operon. To avoid this problem, the plasmid incorporated the endogenous promoter of the operon containing *tonB1* (i.e., prom584) upstream of *HP0581* (*HPB8_778*), the first gene downstream of *tonB1* (Fig. 5B). Transformations of *H. pylori* with this plasmid yielded chloramphenicol-resistant colonies. PCR screening showed that all the chloramphenicol-resistant transformants arose through single-crossover events (leading to integration of the plasmid), rather than double-crossover events (leading to replacement of genomic sequences). These single-crossover events failed to abrogate expression of the *tonB1* gene, as indicated by RT-qPCR analyses (data not shown). Multiple attempts, with or without iron supplementation, were made to generate a Δ*tonB1* mutant, but mutants resulting from double-crossover events were never recovered.

To further evaluate the apparent essentiality of the *tonB1* gene, we introduced a second copy of *tonB1* into the intergenic region between *efp* and *neu* genes (Fig. 5C) and carried out the same mutagenesis protocol to delete the endogenous *tonB1* locus (Fig. 5B). We found that in the presence of a second copy of *tonB1* (i.e., in the *efp-neu* intergenic region), we were now able to successfully delete the endogenous *tonB1* gene. These results indicate that *tonB1* is an essential gene in *H. pylori*.

### Identification of TonB1, TonB2, and TonB3/TolA protein complexes

TonB proteins form a multi-subunit protein complex with two inner membrane proteins (ExbB, ExbD), and ExbBD functions as a rotary motor that is fueled by the inner membrane’s electrochemical gradient. Three TonB-encoding genes (*tonB1*, *tonB2*, *tonB3/tolA*) are annotated in the *H. pylori* B8 genome (36) (Fig. 1). In *H. pylori* strains 26695 and B8, three paralogs each of *exbB* and *exbD* are predicted (Fig. 1; Table 1) (36). *exbB2* (*HP1339*, *HPB8_140*) and *exbD2* (*HP1340*, *HPB8_139*) are in a predicted operon with *tonB2* (*HP1341*, *HPB8_138*) (48). *exbB3* (*HP1445, HPB8_91*) and *exbD3* (*HP1446, HPB8_90*) (Table 1) are in a predicted operon that does not contain a gene encoding TonB (48). Genes encoding ExbB1/TolQ (*HP1130, HPB8_371*) and ExbD1/TolR (*HP1129, HPB8_372*) (Table 1) are in a predicted operon along with *HP1128/HP1127* (*HPB8_373* in B8 nomenclature), which is designated *tonB3/tolA* (Fig. 1) (48). AlphaFold 3 predictions indicate structural similarity among all three ExbB paralogs (Fig. S3A), as well as structural similarity among the three ExbD paralogs (shown for the whole protein in Fig. S3B and C-terminal region of ExbD in panels C and D).

A BLAST analysis of TonB3/TolA (HPB8_373) yielded matches to both TonB and TolA proteins. The predicted structure for TonB3/TolA comprises two helical domains (Fig. S2A) instead of the single helical structure predicted in both TonB1 and TonB2 proteins (Fig. 2A). Interestingly, the predicted structure of TonB3/TolA (Fig. S4A) is also different from canonical TolA proteins in *E. coli* (Fig. S4B) and *Pseudomonas aeruginosa* (Fig. S4C). In addition, unlike TonB1 and TonB2, the TonB3/TolA amino acid sequence contains very few proline residues in the region that forms the periplasmic-spanning flexible spacer domain (Fig. S2C).

The presence of multiple *tonB* paralogs, as well as multiple *exbB* and *exbD* paralogs, suggested that specific combinations of ExbB-ExbD-TonB might assemble into protein complexes. To examine this possibility, we engineered *H. pylori* strains to produce TonB proteins containing HA epitope tags in the regions of the protein predicted to form the flexible spacer (Fig. 2E and F). DNA sequences for these HA-tagged proteins were inserted into the intergenic region between *neuB* and *efp* genes. To ensure the formation of only HA-tagged TonB complexes, the wild-type endogenous TonB locus was deleted in the respective strains engineered to produce HA-tagged TonB proteins. HA-tagged TonB2 strains (i.e., JL1028, Table 2) were recovered in the absence of supplemental iron, which indicated that the HA tag in TonB2 did not disrupt its function. Similarly, the introduction of the HA tag into TonB1 did not disrupt the function of TonB1, as we were able to recover HA-TonB1-producing strains in which the endogenous copy of *tonB1* was deleted (i.e., JL1031, Table 2). In addition to the two TonB-tagged strains, we generated a strain (i.e., JL1033, Table 2) engineered to produce HA-tagged TonB3/TolA (Fig. S2B).

The TonB1 HA-tagged (JL1031), TonB2 HA-tagged (JL1028), TonB3/TolA HA-tagged (JL1033), and wild-type strains (as an additional control) were cultured under the same routine conditions, the epitope-tagged proteins were immunopurified, and the resulting preparations were analyzed by mass spectrometry, as described in the “Materials and Methods” section. As shown in Table 3, high levels of both ExbD2 and ExbB2 are detected in the immunopurified fractions of JL1028, a strain expressing TonB2-HA. In contrast, very low levels of ExbB1/TolQ, ExbB3, or ExbD1/TolR, ExbD3 were detected in the TonB2 pull-down assays. Importantly, little to no ExbB2 or ExbD2 was detected in pull-down assays involving JL1033 (i.e., TonB3/TolA-HA) or the wild-type strain. This result suggested a high level of specificity in the interaction of TonB2 with ExbB2 and ExbD2. Consistent with this, in a previous study, we found that ExbB2 and TonB2 co-purified with FLAG-tagged ExbD2, and there was no significant co-purification of other ExbB, ExbD, or TonB proteins (57) (Table S1). Control experiments with LolF-FLAG or FrdC-FLAG did not result in co-purification of TonB, ExbB, or ExbD proteins (57) (Table S1). Co-purification of TonB2, ExbB2, and ExbD2 in these experiments is consistent with the observation that TonB2, ExbB2, and ExbD2 are encoded by genes predicted to be transcribed from the same operon (designated *HP1339-HP1340-HP1341* in *H. pylori* strain 26695) (Fig. 1).

**TABLE 3:** LC-MS/MS analysis of proteins immunopurified from *H. pylori* strains harboring HA-TonB1, HA-TonB2, and HA-TonB3/TolA

Experiment 1						Experiment 2			
Identified protein^a^		Bait protein (HA-TAG)				Bait protein (HA-TAG)			
		No bait^c^^,d^	HA-TonB3/TolA^c^^,d^	HA-TonB2^c^^,d^	HA-TonB1^c^^,d^	No bait^c^^,d^	HA-TonB3/TolA^c^^,d^	HA-TonB2^c^^,d^	HA-TonB1^c^^,d^
Gene number^b^	Name								
HP0582	TonB1	0	1	18	**1,433**	0	2	8	**1,474**
HP1446	ExbD3	9	10	18	**226**	8	8	14	**569**
HP1445	ExbB3	0	0	6	**210**	2	3	4	**221**
HP1341	TonB2	6	11	**813**	35	4	17	**804**	82
HP1340	ExbD2	0	1	**164**	7	2	6	**257**	29
HP1339	ExbB2	4	1	**58**	2	4	0	**136**	5
HP1127/HP1128	TonB3/TolA	0	**1,308**	45	17	5	**1,765**	74	15
HP1129	ExbD1/TolR	1	**70**	6	0	1	**113**	5	0
HP1130	ExbB1/TolQ	1	**383**	18	1	3	**809**	153	43
HP0686	FecA1	0	14	7	**67**	1	5	1	**80**
HP0807	FecA2	0	0	0	**17**	0	0	0	**30**
HP1400	FecA3	4	26	21	**102**	45	18	2	**89**
HP0876	FrpB1	0	0	1	**12**	0	0	1	**12**
HP0915/916	FrpB2/3	0	0	0	0	0	0	0	0
HP1512	FrpB4	25	113	57	**262**	32	83	16	**180**
HP1126	TolB	1	**131**	0	12	8	**143**	4	21
HP1125	Pal	14	38	20	40	9	44	7	38
TonB-related spectral counts		50	1,938	1,232	2,391	107	2,829	1,475	2,829
% TonB-related counts		0	12.1	6.2	14.4	0.7	14.5	7.0	16.7
Total spectral counts		10,927	16,039	19,968	16,616	15,986	19,459	20,948	16,960

^a^ TonB-related proteins identified by MS.

^b^ Gene numbers according to *H. pylori* 26695 genome.

^c^ Immunopurifications were performed as described in the Methods for *H. pylori* strains harboring the indicated HA tags. Strains JL1028, JL1031, and JL1033 (Table 2) were used for immunopurification of TonB2-HA, TonB1-HA, and TonB3/TolA-HA complexes, respectively. No bait corresponds to wild-type strain 7.13.

^d^ Spectral counts for the listed proteins are indicated. Bold numbers indicate proteins that are specifically immunopurified by the indicated HA Tag.

Pull-down assays with the TonB1-HA-tagged strain JL1031 yielded ExbB3 and ExbD3 (Table 3). Very low levels of ExbB1/TolQ, ExbB2, ExbD1/TolR, and ExbD2 were detected. Moreover, control pull-downs with the WT strain or the TonB3/TolA-HA-producing strain JL1033 did not yield ExbB3 or ExbD3 (Table 3). These findings provide evidence that TonB1 specifically interacts with ExbB3 and ExbD3.

The TonB3/TolA-HA pull-down assays with strain JL1033 specifically pulled down ExbD1/TolR and ExbB1/TolQ, but not ExbB2, ExbB3, or ExbD2, ExbD2 (Table 3). Control immunoprecipitation pull-downs of the WT (untagged) strain did not pull down ExbD1/TolR or ExbB1/TolQ.

Figure S5 summarizes the interactions of the TonB proteins with ExbB and ExbD complexes. These results provide evidence for the assembly of three unique TonB complexes in *H. pylori*: TonB1-ExbB3-ExbD3, TonB2-ExbB2-ExbD2, and TonB3/TolA-ExbB1/TolQ-ExbD1/TolR.

In addition to interactions of TonB proteins with the inner membrane complex ExbBD, TonB proteins are also expected to interact with specific TBDTs localized to the outer membrane of *H. pylori*. As shown in Table 3, spectral counts for 5 of the 6 TBDTs were identified following TonB1-HA immunoprecipitation using strain JL1031. Spectral counts for FecA1, FecA2, FecA3, FrpB1, and FrpB4 were present in the TonB1-HA immunoprecipitation but were absent or at very low levels in immunoprecipitations with the control (untagged) WT strain.

### TonB3/TolA-HA interacts with TolB protein

As shown in Table 3, TolB co-purified with TonB3/TolA-HA in the pull-down assays using strain JL1033. Little to no TolB was detected in the other immunoprecipitation assays involving WT, TonB1-HA (JL1031), or TonB2-HA (JL1028)-producing strains. These results provide evidence for a specific interaction of TonB3/TolA with TolB (Fig. S5C). This finding suggests that TonB3/TolA may be a member of the Tol system. Tol systems are similar to the TonB system in that both systems harvest the proton-motive force of the inner membrane to carry out their respective outer membrane functions (27, 58, 59). TolB is predicted to interact with Pal (32, 58, 59), but TonB3/TolA pull-down assays revealed only a slightly increased level of Pal compared to pull-down assays with TonB1-HA, TonB2-HA, and WT strains (Table 3).

To evaluate if *tonB3/tolA* is an essential or non-essential gene, mutagenesis of *HP1128/HP1127* (designated *HPB8_373* in *H. pylori* strain B8) was carried out as described in the “Materials and Methods” section, replacing the *tonB3/tolA* gene with an *aph3* cassette. TonB3/TolA mutants, such as JL1032 (Table 2) were obtained on a routine basis, without a requirement for supplemental metals.

To further explore the possibility that HPB8_373 (TonB3/TolA) may be a Tol protein, we examined the genomic organization of the genes surrounding TolB in multiple bacterial species, focusing on the organization of genes that comprise the Tol system (KEGG Pathway Database). Figure S6 illustrates that members of the Tol system follow a gene order of *tolQ*, *tolR*, *tolA*, *tolB,* and *pal*, an organization that is well conserved among gamma-, beta-, alpha-, and delta-Proteobacteria. In Campylobacteriota (formerly known as epsilon-Proteobacteria), such as *H. pylori* and *Campylobacter*, *exbB1/tolQ*, *exbD1/tolR,* and *tonB3/tolA* are the genes that precede *tolB* and *pal* (Fig. S6), raising the possibility that in *H. pylori*, the annotated *exbB1/tolQ*, *exbD1/tolR,* and *tonB3/tolA* genes may actually encode proteins corresponding to TolQ, TolR, and TolA, respectively. Indeed, the AlphaFold 3-predicted structure of TonB3/TolA (Fig. S2A) is different from that of TonB1 and TonB2 (Fig. 2A). Moreover, a BLAST search of *HPB8_373* (encoding TonB3/TolA in *H. pylori* strain B8) yielded several hits predicting TonB3 to be a TolA protein. While the overall predicted structure of *H. pylori* TonB3/TolA differs substantially from structures of *E. coli* and *Pseudomonas* TolA (Fig. S4), a comparison of the C-terminal domains of the three proteins did show structural similarities (Fig. S4D).

In addition to analyzing the AlphaFold 3-modeled structure of HPB8_373 (TonB3/TolA), we compared the modeled structures of ExbB1/TolQ and ExbD1/TolR from *H. pylori* strain B8 with the respective *E. coli* TolQ and TolR orthologs. As illustrated in Fig. S7 and S8, AlphaFold 3-modeled *H. pylori* ExbB1/TolQ and ExbD1/TolR exhibit a high level of structural similarity to *E. coli* TolQ and TolR, respectively. Consistent with this observation, a previous study in *E. coli* reported that ExbB and ExbD can partially substitute for the functions of TolQ and TolR, and conversely, TolQ and TolR can partially replace ExbB and ExbD (60–62)

## DISCUSSION

While the core architecture of TonB energy transduction systems is well characterized, extensive variation exists in the structural organization, functional roles, and substrate specificities of TonB systems across different Gram-negative bacterial species. For instance, *Escherichia coli* K-12 harbors a single TonB-ExbB-ExbD complex that interfaces with seven TonB-dependent transporters (TBDTs), six of which facilitate uptake of ferric iron-chelated siderophores (63), with one mediating transport of vitamin B12 (64). In contrast, other bacterial species (e.g., *Bacteroides thetaiotaomicron, Riemerella anatipestifer*) possess multiple TonB homologs, which exhibit either specialized functions or functional redundancy (33, 35, 65).

Beyond TonB diversity, variation in associated ExbB and ExbD proteins further contributes to functional heterogeneity. Previous studies have detected specific protein-protein interactions and complex formation among these components in several species. In *R. anatipestifer*, TonB1 associates selectively with ExbB1 and ExbD1, forming a discrete TonB1-ExbB1-ExbD1 complex, while TonB2 similarly assembles into a TonB2-ExbB2-ExbD2 complex (35). In *Vibrio cholerae*, the TonB1-ExbB1-ExbD1 system facilitates hemin transport (66, 67), whereas the TonB2-ExbB2-ExbD2 system is important for uptake of the siderophore enterobactin (67, 68).

TBDT gene content also varies markedly across bacterial species, reflecting adaptation to ecological niches and the specific nutritional requirements of each organism. The number of TBDTs ranges from 7 in *E. coli* (63) to over 100 in *Bacteroides* species (69), with *Caulobacter crescentus* encoding up to 65 distinct TBDTs (70, 71). This genomic variability underscores the flexibility of TonB systems in mediating substrate acquisition.

The genome annotation of *Helicobacter pylori* strain B8 (36) designates three TonB proteins, three ExbB proteins, three ExbD proteins, and six TonB-dependent transporters (TBDTs). Interestingly, there is variation in the number of *tonB* orthologs reported in the genome annotations of different *H. pylori* strains. For example, genes for all three *tonB* paralogs (*tonB1*, *tonB2*, *tonB3/tolA*) are present in *H. pylori* strains J166, G27, B8, J99, 26695, and P12 (23, 24, 36–38, 47). However, only two (i.e., *tonB1* and *tonB2*) are annotated in strain J99 (24), and only *tonB2* is annotated in strain 26695 (23) and strain P12 (47). These variations might reflect incomplete or inaccurate genome annotations or differences in the algorithms used for annotation.

In the current study, we show that the three *H*. *pylori tonB* genes in *H. pylori* 7.13 (*tonB1, tonB2, tonB3/tolA*) exhibit varying degrees of essentiality. TonB1 mutants are non-viable, while TonB2 mutants can survive only when supplemental iron is provided. In contrast, TonB3/TolA mutants show no impairment in their ability to grow *in vitro*. Structural modeling reveals that TonB1 and TonB2 exhibit a high degree of similarity.

Through immunopurification experiments, we found that TonB2 co-purified with ExbB2 and ExbD2. This relationship is consistent with the clustering of all three genes (*tonB2*, *exbB2*, and *exbD2*) in an operon. A previous study (22) reported that a *tonB2* mutant was defective in urease activity and the transport of nickel, an essential cofactor of the urease enzyme. Our current data are consistent with these findings. A previous study demonstrated that the difference in urease activity and nickel content between WT and the *tonB2* mutant was more pronounced when the bacteria were grown under low pH conditions (22). Similarly, we found that differences in urease activity between the WT and *tonB2* mutant were most prominent when cultures were grown under low pH conditions (data not shown). The previous study (22) reported that FrpB4 was also involved in nickel transport. While we detected co-purification of FrpB4 with TonB2, this association was not very striking under the pH 7.0 growth conditions examined.

Immunopurification experiments showed that TonB1 co-purifies with ExbB3 and ExbD3, suggesting that these are components of a protein complex. Additionally, TonB1 co-purified with five of the six predicted TBDTs (FecA1, FecA2, FecA3, FrpB1, and FrpB4). The remaining TBDT (designated FrpB2/3) did not co-purify with any of the three TonB proteins and is likely non-functional, as the gene is fragmented into two separate open reading frames in *H. pylori* strain 7.13 (used for immunoprecipitation experiments) and multiple other *H. pylori* strains (strains B8 and 26695). The association of TonB1 with multiple TBDTs may account for the inability to generate a TonB1-specific knockout mutant, as disruption of TonB1 might impair uptake of multiple nutrients required for *H. pylori* viability.

In the *H. pylori* B8 genome, *tonB3/tolA* has been annotated as a third TonB homolog (36). Several features of TonB3 suggest that it might be a component of the Tol system instead of a canonical TonB-dependent transport pathway. While the Ton system is primarily involved in nutrient transport, the Tol system functions to maintain outer membrane integrity and facilitate cell division (32, 58, 72, 73). The Tol system consists of five core proteins: TolQ, TolR, TolA, TolB, and Pal (28, 58, 59, 61). TolQ, TolR, and TolA form an inner membrane energy-harvesting complex analogous to ExbB-ExbD-TonB in the Ton system (59–62). The energized TolA interacts with TolB and triggers the release of Pal, which anchors the peptidoglycan cell wall to the outer membrane, thereby stabilizing cellular architecture.

In this study, TonB3/TolA co-purified with ExbB1/TolQ and ExbD1/TolR and TolB. Genomic analysis of the region flanking the *tonB3/tolA* locus revealed an organization reminiscent of canonical Tol systems found in Gram-negative bacteria (Fig. S6). The relative positioning of *exbB1/tolQ*, *exbD1/tolR*, and *tonB3/tolA* upstream of *tolB* and *pal* suggests that these genes may encode functional analogs of TolQ, TolR, and TolA, respectively. In *E. coli*, TolQ and TolR have 50% sequence similarity with ExbB and ExbD, respectively, and the proteins from the different systems are able to partially cross-complement each other (62). Moreover, the modeled architectures of *H. pylori* ExbB1/TolQ and ExbD1/TolR closely resemble those of *E. coli* TolQ and TolR (Fig. S7 and S8). In contrast, *H. pylori* TonB3/TolA did not appear to be structurally similar to TolA proteins from *E. coli* or *P. aeruginosa*. Nonetheless, its interaction with TolB and its location upstream of TolB suggest that it may in fact represent a functional TolA analog.

In summary, at the outset of this study, it was unclear how the multiple paralogs of *H. pylori* TonB, ExbB, ExbD, and TBDTs were physically or functionally related, and the functions of most of these proteins had not been investigated in detail. The results reported in this study reveal the existence of three distinct protein complexes involving these proteins. We show that *H. pylori tonB1* is an essential gene, *tonB2* mutants require supplemental iron for growth, and *tonB3*/*tolA* is non-essential for growth *in vitro*. In future studies, it will be important to further elucidate the functions of these three systems, especially in the context of *H. pylori* infection. For example, while *tonB3/tolA* is non-essential for *H. pylori* growth *in vitro*, it may contribute to *H. pylori* colonization of the stomach.

## MATERIALS AND METHODS

### Bacterial culture methods

*H. pylori* strains analyzed in this study are listed in Table 2. *H. pylori* strains were grown at 37°C in ambient air supplemented with 5% CO_2_ on Trypticase Soy Agar plates containing 5% sheep blood or in bisulfite-free *Brucella* broth containing 5% fetal bovine serum (FBS) (i.e., BB-FBS). When necessary, streptomycin (25 μg/mL), chloramphenicol (5 μg/mL), gentamicin (10 µg/mL), or kanamycin (10 μg/mL) was added to the culture medium. *Escherichia coli* was grown on Luria-Bertani medium in the presence of ampicillin (50 μg/mL), chloramphenicol (25 μg/mL), streptomycin (25 μg/mL), or kanamycin (25 μg/mL).

### Generation of *tonB* mutant strains

We used *H. pylori* 7.13 (44), a strain capable of causing gastric cancer in a Mongolian gerbil model, for the generation of *tonB* mutants. We utilized the published genome sequence of strain B8 (a closely related strain) (36) as a framework to design plasmids (Table 2) to generate *tonB* mutants in strain 7.13. The genetic organization of *tonB* paralogs and related genes is identical in whole-genome sequences for *H. pylori* 7.13 and B8 strains.

The *tonB2* gene is the third gene in an operon containing *exbD2*, *exbB2*, and *tonB2* (Fig. 1). To generate a *tonB2* mutant (JL1025), a *tonB2* deletion plasmid (p1341kan, Table 2) was synthesized by Genscript. This plasmid contained an *aph3* cassette (encoding kanamycin resistance) flanked by nucleotides present 500 bp upstream and downstream of the *tonB2* gene, cloned into the pUC57 plasmid, a high-copy-number cloning vector containing the pMB1 origin of replication. This plasmid, which does not replicate in *H. pylori*, was used to transform *H. pylori* strain 7.13, and transformants resulting from double-crossover homologous recombination events were selected on *Brucella* plates containing 10 μg/mL kanamycin. A previous study (22) reported that *tonB2* mutants were only isolated under conditions of iron supplementation. Based on this, 1 mM FeCl_3_ was added to transformation plates. Genomic preps were screened with primers 1341 screenfor (5′-TTCTAACGCGCCTTATGTGGG-3′) and 1341 screenrev (5′-CCAAGATTGGGGCCAGGG-3′), and the presence of the expected mutations was confirmed by DNA sequencing. Strain JL1025 (Table 2) was chosen for further analysis.

The *tonB3/tolA gene* (designated *HP1128/HP1127* in *H. pylori* strain 26695 and *HPB8_373* in *H. pylori* strain B8) is predicted to be the tenth gene in a large operon beginning with gene HP1137 (48) (Fig. 1). The pUC57-based plasmid p1128/1127 kan (Genscript) (Table 2), which does not replicate in *H. pylori*, contains a kanamycin cassette flanked by DNA sequences present 500 bp upstream and downstream of the *tonB3/tolA* gene. The 500 bp downstream includes the ORF for HP1126 (*tolB*). To ensure that the *tonB3/tolA* deletion did not affect transcription of genes downstream of *tonB3/tolA*, plasmid p1128/1127 kan was designed to include the promoter sequence for *HP1137* upstream of *HP1126* (48). The *tonB3/tolA* mutants were generated by transforming *H. pylori* strain 7.13 with the non-replicating p1128/1127 kan plasmid and selecting transformants on Brucella plates containing 10 µg/mL kanamycin. This resulted in the isolation of the *tonB3/tolA* mutant strain JL1032 (Table 2). No supplemental iron was necessary to obtain the *tonB3/tolA* mutant strain.

In *H. pylori* strain 26695, the *tonB1* gene is in a large operon extending from *HP0584 to HP0578* (i.e., *HPB8_781* to *HPB8_774* in the *H. pylori* B8 genome) (Fig. 5) (48). To mutate *tonB1*, pUC57-based plasmid p582cat (Genscript; Table 2) was designed to replace *tonB1* (i.e., HP0582) with a *cat* (chloramphenicol acetyltransferase) cassette. To allow for homologous recombination, the *cat* cassette was flanked by nucleotide sequences present 500 bp upstream (containing *HP0583*) and downstream of the *tonB1* gene (containing *HP0581*). To ensure continued expression of genes downstream of *HP0582*, plasmid p582cat was also designed to place the promoter of *HP0584* upstream of the *HP0581* ORF, thereby driving transcription of genes *HP0581* to *HP0578* (Fig. 5). p582cat was used to transform strain 7.13, and transformants were selected in the presence or absence of supplemental iron. PCR screening of chloramphenicol-resistant colonies with primers 582screenfor (5′-gcacggacgctagagtgaat-3′) and 582screenrev (5′-aaagggggcttttggaggag-3′) indicated the occurrence of single-crossover events, and RT-qPCR analyses using *tonB1* specific primers 5′-cagtaaaaacaacccgggcg-3′ and 5′-tgggctttttgggttctggt-3′ revealed sustained *tonB1* expression in the mutant strains.

### Construction of strains producing HA-tagged TonB proteins

To generate strains producing an HA-tagged TonB2 protein, the pUC57-based plasmid (ptonB2 HA, Table 2) was synthesized by Genscript. This non-replicating plasmid was designed to introduce, by homologous DNA recombination into the *H. pylori* genome, sequences that placed a HA tag (YPYDVPDYA) immediately after proline 134 in the proline-rich rod structure of TonB2. The plasmid ptonB2 HA also contained nucleotide sequences that allowed introduction of the gene encoding HA-tagged TonB2 into the intergenic region between *neuB* and *efp* genes (corresponding to *HP0177* and *HP0178* in *H. pylori* strain 26695 [23] and *HPB8_1388* and *HPB8_1390* in *H. pylori* strain B8) (36). To introduce sequences encoding the HA-tagged *tonB2* gene into the intergenic region between *neuB* and *efp* genes as an unmarked mutation, a previously described counterselection method using an *aacC4-rpsL* cassette was employed (54). This cassette confers resistance to gentamicin, mediated by the aminoglycoside-3-acetyltransferase IV (*aacC4*) gene, and susceptibility to streptomycin, mediated by the intact *rpsL* gene from *H. pylori* 26695. As a first step in the counterselection mutagenesis, *rpsL* mutants were generated by transforming the Δ*tonB2::aph3* mutant strain JL1025 with a non-replicating plasmid (pTB2, Table 2) containing a cloned *H. pylori rpsL* gene harboring an A-to-G mutation at codon 43 of *rpsL* (55, 56). The resultant Lys (K)-to-Arg (R) amino acid substitution at position 43 of RpsL confers streptomycin resistance to *H. pylori* strains containing this mutation. The streptomycin-resistant *rpsL-K43R* JL1025 mutants were next transformed with p*177-aacC4-rpsL* (Table 2), a non-replicating plasmid that allows the insertion of an *aacC4-rpsL* cassette (conferring gentamicin resistance) (74) into the intergenic region between genes *HPB8_1388* (*neuB*) and *HPB8_1390* (*efp*). Colonies that were resistant to gentamicin (10 µg/mL) and sensitive to streptomycin (25 µg/mL) were selected, and proper insertion into the *neuB-efp* locus was confirmed by PCR. The resultant strain JL1026 (*rpsL-K43R*, *ΔtonB2::aph3*, *neuB-efp::aacC4-rpsL*) was next transformed with ptonB2 HA (Table 2), resulting in a streptomycin-resistant strain (JL1028, Table 2) expressing an HA-tagged TonB2 protein. JL1026 was also used to generate a complemented *tonB2* strain (JL1027, Table 2) when transformed with ptonB2 (Table 2), a plasmid identical to ptonB2 HA but lacking the HA tag.

A similar counterselection method was used to create strains producing an HA-tagged TonB1 protein. However, the inability to generate a *tonB1* deletion mutant (described above) required the initial introduction of a gene encoding HA-TonB1 into the intergenic region between the *neuB* and *efp* genes of wild-type 7.13 strain, using the methods described above for TonB2-HA. Briefly, *H. pylori* strain 7.13 was transformed with plasmid pTB2, a non-replicating plasmid containing a cloned *H. pylori rpsL* gene harboring a lysine-to-arginine mutation at codon 43 of *rpsL*, conferring streptomycin resistance to strain 7.13 (55, 56). The resulting strain was transformed with plasmid *p177-aacC4-rpsL* to create JL1029. JL1029 was next transformed with the non-replicating pUC57-based plasmid pTonB1 HA (synthesized by Genscript; Table 2) to create JL1030, which expresses TonB1-HA from the *neuB-efp* locus. pTonB1 HA was designed to introduce the HA-tag after amino acid 99 (threonine) in the proline-rich rod structure of TonB1. We next deleted the endogenous *tonB1* locus by transforming JL1030 with plasmid p582cat (Fig. 5B; Table 2). Chloramphenicol-resistant colonies PCR-amplified with primers 582screenfor/rev yielded the expected PCR fragments, and the expected sequences were confirmed by DNA sequencing in strain JL1031 (Table 2).

To create TonB3/TolA HA-tagged strains in *H. pylori*, plasmid pTonB3 HA-CAT (Genscript) (Table 2), encoding HA-tagged TonB3/TolA (with the HA tag located after E81 in the unstructured region of TonB3/TolA) and a chloramphenicol (*cat*) cassette (used as an antibiotic selection marker), was used to transform the *tonB3/tolA* mutant strain JL1032 (Table 2), creating strain JL1033 (Table 2). The non-replicating plasmid pTonB3 HA-CAT (Table 2) contained DNA sequences that allowed the introduction of DNA sequences encoding TonB3/TolA HA and CAT into the *neuB-efp* locus in strain JL1033.

The proper introduction of sequences encoding HA tags into strains JL1028 (TonB2-HA), JL1031 (TonB1-HA), and JL1033 (TonB3-HA) was confirmed by PCR amplification of the *neuB-efp* locus using primers 177screen (5′-tgcgaagagcctaatctggt-3′) and 177screenrev (5′-acagcgcttttagcatggat-3′) followed by DNA sequencing of the PCR products.

To verify the production of HA-tagged proteins, immunoprecipitated protein samples from JL1028 (TonB2-HA), JL1031 (TonB1-HA), and JL1033 (TonB3-HA) were analyzed by silver staining and Western blotting. Silver staining revealed the presence of protein bands in the purified samples from JL1028 (TonB2 HA), JL1031 (TonB1 HA), and JL1033 (TonB3/TolA HA) that were absent in the WT samples. Importantly, the protein bands were of the expected molecular weights for the TonB-tagged proteins (data not shown). In addition, Western blotting with an anti-HA antibody revealed immunoreactive bands with the expected molecular weights for each of the HA-tagged proteins (data not shown).

### Immunopurification of *H. pylori* proteins

*H. pylori* strains were grown in bisulfite-free *Brucella* broth containing 10% FBS (BB-FBS; 125 mL cultures) for 24 h, resulting in optical density values at 600 nm (OD_600_) between 0.6 and 0.9. The bacterial cells were then pelleted at 5,000 × *g* for 10 min. The bacterial pellets were next resuspended in RIPA buffer (0.2 M HEPES, 0.3 M NaCl, 1% NP-40, 0.25% sodium deoxycholate, pH 7.0) supplemented with 1 mM phenylmethylsulfonyl fluoride (PMSF) and a protease inhibitor cocktail (cOmplete Mini, Roche). The bacterial suspensions were sonicated on ice (25% amplitude with four pulses, 10 s on/10 s off, using a 1/8 inch probe) and incubated for 1 h at 4°C. The bacterial lysates were then centrifuged (12,000 × *g* for 15 min at 4°C) to pellet insoluble material. Monoclonal anti-HA antibodies non-covalently linked to protein G Dynabeads (Invitrogen) were then incubated with the clarified bacterial lysate for 30 min at room temperature. Following three washes of the beads with RIPA wash buffer (with reduced amount of sodium deoxycholate (0.025%), proteins bound to the beads were selectively eluted with RIPA wash buffer containing 200 µg/mL HA peptide (YPYDVPDYA; GenScript).

### Immunoblotting

Protein lysates were separated by SDS-PAGE and transferred to 0.22 μm nitrocellulose membranes (Li-Cor). Membranes were blocked in 2% non-fat dry milk in tris-buffered saline containing 0.1% Tween-20. Target proteins were detected by incubating membranes with a mouse monoclonal antibody against HA (12CA5, 1:5,000 dilution) (75, 76), followed by a horseradish peroxidase-conjugated secondary anti-mouse antibody. Signal detection was performed using enhanced chemiluminescence (ECL).

### ICP-MS analyses

*H. pylori* cultures were grown in metal-free conical tubes (VWR), with growth conditions as described in the relevant experiments (see figure legends for details). Supplemental iron was required for *H. pylori* Δ*tonB2* mutant growth. Therefore, overnight cultures grown in 25 µM FeCl_3_ were washed, and *H. pylori* cells were subcultured into medium containing a lower amount of iron (i.e., 5 µM of FeCl_3_) for 7 h. This lower concentration (5 µM FeCl_3_) was sufficient to allow continued growth of the *H. pylori* Δ*tonB2* mutant over the 7 h time period, prior to cells being harvested for ICP-MS analyses. Cells for ICP-MS analyses were fixed with acetone:EtOH for 3 min prior to being pelleted by centrifugation.

Transition metals were quantified by inductively coupled plasma mass spectrometry (ICP-MS). Samples were digested in metal-free 15 mL conical tubes in 200 µL of 70% Optima-grade nitric acid at 65°C overnight, then diluted with UltraPure water to 2% nitric acid for analysis. Elemental quantification was conducted using an Agilent 7700 ICP-MS attached to an ASX-560 autosampler. The settings for analysis were cell entrance = −40 V, cell exit = −60 V, plate bias = −60 V, OctP bias = −18 V, and helium flow = 4.5 mL/min. Optimal voltages for extract 2, omega bias, omega lens, OctP RF, and deflect were empirically determined. Calibration curves for elements were generated using ARISTAR ICP standard mix. Samples were introduced by peristaltic pump with 0.5-mm-internal-diameter tubing through a MicroMist borosilicate glass nebulizer. They were initially taken up at 0.5 rps for 30 s, followed by 30 s at 0.1 rps to stabilize the signal. Spectrum mode analysis was performed at 0.1 rps, collecting three points across each peak and conducting three replicates of 100 sweeps for each element. The sampling probe and tubing were rinsed with 2% nitric acid for 30 s at 0.5 rps between each sample. Data were acquired and analyzed using Agilent MassHunter Workstation, software version A.01.02. Individual ion concentrations are reported relative to the ^34^S concentration in the sample to control for total cell abundance.

### Urease assay

To assess urease activity, a solution containing 2% urea in 0.5 M sodium phosphate buffer (pH 7.0) was prepared, followed by the addition of phenol red. The assay solution exhibited colorimetric changes in response to variations in pH. To evaluate urease function, overnight cultures of WT, JL1025 (Δ*tonB2::aph3*, Table 2)*,* and JL1027 (complemented *tonB2* strain; Table 2) grown at pH 7.0 and supplemented with 25 µM FeCl_3_ were subcultured into pH 5.3 medium containing 5 µM FeCl_3_ buffered with MES [2-(N-morpholino) ethanesulfonic acid]. After incubating for 2 h, the subcultured cells were introduced into the urease assay solution (0.5 M sodium phosphate buffer, pH 7.0 containing 2% urea). Following a 30-minute reaction period, the absorbance of the solution was measured at OD_540_ nm. Urease activity was then normalized to the OD_600_ of the bacterial cultures.

## SUPPLEMENTAL MATERIAL

Supplemental figures iai.00018-26-s0001.pdf Fig. S1 to S8.

Supplemental material iai.00018-26-s0002.pdf Supplemental figure legends.

Table S1 iai.00018-26-s0003.pdf LC-MS/MS analysis of proteins immunopurified from *H. pylori* strains harboring ExbD2-FLAG, LolF-FLAG, FrdC-FLAG.

ASM does not own the copyrights to Supplemental Material that may be linked to, or accessed through, an article. The authors have granted ASM a non-exclusive, world-wide license to publish the Supplemental Material files. Please contact the corresponding author directly for reuse.
