# Supplementary material for: Multiple TonB-dependent transport systems in Helicobacter pylori
Source: Infect Immun. 2026 Jun 4;94(7):e00018-26. doi: 10.1128/iai.00018-26 (PMC13367063; doi:10.1128/iai.00018-26)
Supplement: Supplemental figures — Fig. S1 to S8. [file iai.00018-26-s0001.pdf]

```
TonB1           MPENSKLQPAKLGKNFDPVDHSNRNFFFSLILSVLLHWLIYFLFEHREDFFPSKPKLVKL
TonB2           -----------MKISPSPRKLSKVSTSVSFLISFALYAIGFGYFLLREDAP--EPLAQAG
                           :  . .* . *: .  .*:::*. *: : :  *  ***    :*

TonB1           NPENLLVLKRGHSQDPSKNTQGAPKPTLAGPQKPP-TPPTPPTPPTPPTPPKPIEKPKPE
TonB2           TTKVTMSLASINTNSNTKTNAESAKPKEEPKEKPKKEEPKKEEPKKEVTKPKPKPKPKPK
                ..:  : *   :::. :*..  :.**.    :**    *.   * .  * ***  ****:

TonB1           PKPKPKPEPK-KPNHKHKALKKVEKVEEKKVVEEKKEEKKIVEQKVEQKVEQKKIEEKKP
TonB2           PKPKPKPEPKPEPKPEPKPEPKVEEVKKEEPKEEPKKEE--AKEEAKEKSAPKQVTTKDI
                ********** :*: : *. ***:*::::   ** *:*:  .::.::*    *::  *.

TonB1           VKKEFDPNQLSFLPKEVAPPRQENNKGLDNQTRRDIDELYGEEFGDLGTAEKDFIRNNLR
TonB2           VKEKDKQEESNKTSEGATSEAQAYNPGVSNEFLMKIQTAISSKN----------------
                **:: . :: .  .: .:.  *  * *:.*:   .*:   ..:

TonB1           DIGRITQKYLEYPQVAAYLGQDGTNAVEFYLHPNGDITDLKIIIGSEYKMLDDNTLKTIQ
TonB2           ----------RYPKMAQIRGIEGEVLVSFTINADGSVTDIKVVKSNTTDILNHAALEAIK
                          .**::*   * :*   *.* ::.:*.:**:*:: ..  .:*:. :*::*:

TonB1           IAYKDYPRPKTKTLIRIRVRYYLGGN
TonB2           SAAHLFPKPEETVHLKIPIAYSLKED
                 * : :*:*: .. ::* : * *  :
```

Supplemental Figure 1.

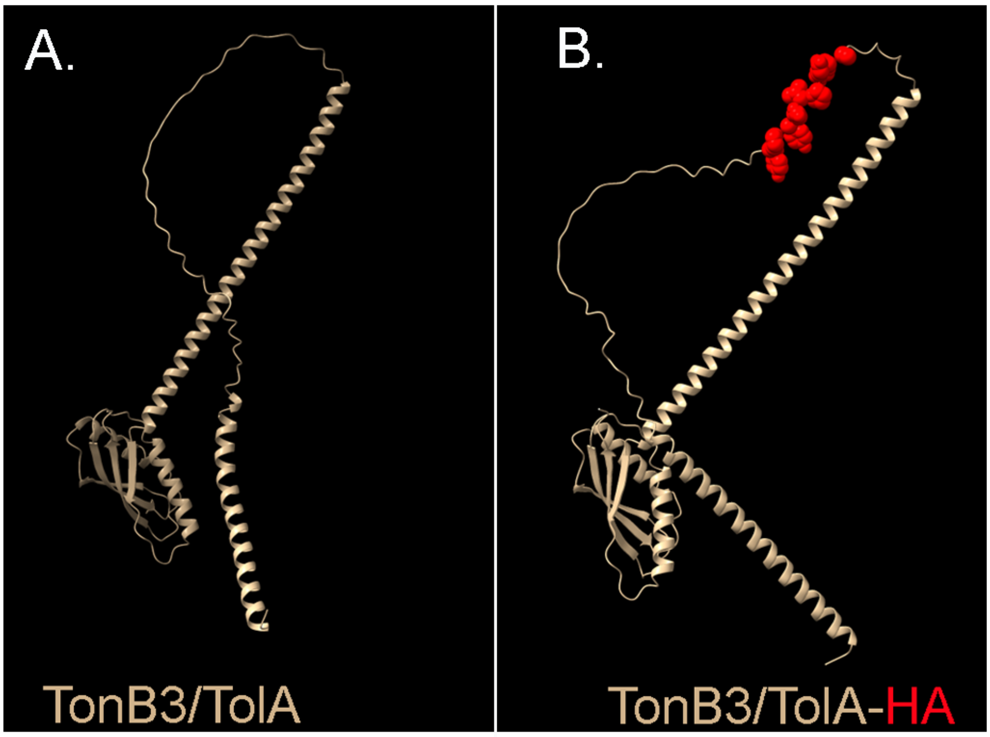

C.

```
TonB1           MPENSKLQPAKLGKNFDPVDHSNRNFFFSLILSVLLHWLIYFLFEHREDFFPSKPKLVKL
TonB2           -----------MKISPSPRKLSKVSTSVSFLISFALYAIGFGYFLLREDAP--EPLAQAG
TonB3/TolA      -----------------MSKSAIFVVSGFLAFLLYALLLYGLLLGRHNKEAEKILLDLG
                                   . *      .::  .    : :  :  *.:    :

TonB1           NPENLLVLKRGHSQDPSKNTQGAPKPTLAGPQKPP-TPPTPPTPPTPPTPPKPIEKPKPE
TonB2           TTKVTMSLASINTNSNTKTNAESAKPKEEPKEKPKKEEPKKEEPKKEVTKPKPKPKPKPK
TonB3/TolA      -----------------KKNEQVIDLNLEDLPSEKKNEKIEKVTEKQGDFLEPKEEPKEE
                                 *..    . .     .          . .     :*  :** :

TonB1           PKPKPKPEPK-KPNHKHKALKKVEKVEEKKVVEEKKEEKKIVEQKVEQKVEQKKIEEKKP
TonB2           PKPKPKPEPKPEPKPEPKPEPKVEEVKKEEPKEEPKKEE--AKEEAKEKSAPKQVTTKDI
TonB3/TolA      PEESLEDIFSSLNDFQEKTDKNAQKDEQKNEQEEQRRLR--EQQRLKQNQENQEMLKGLQ
                *: . :   .   . : *.  :.:: ::::  ** :. .   ::. :::   :::

TonB1           VKKEFDPNQLSFLPKEVAPPRQENNKGLDNQTRRDIDELYGEEFGDLGTAEKDFIRNNLR
TonB2           VKEKDKQEESNKTSEGATSEAQAYNPGVSNEFLMKIQTAISSKN----------------
TonB3/TolA      QNLNQFTQKLESVKNKTLDLQVPKQDGVDEKAYQEWYAQIYQIL----------------
                 : :   :: .   : .       : *:.::   .      .

TonB1           DIGRITQKYLEYPQVAAYLGQDGTNAVEFYLHPNGDITDLKIIIGSEYKMLDDNTLKTIQ
TonB2           ----------RYPKMAQIRGIEGEVLVSFTINADGSVTDIKVVKSNTTDILNHAALEAIK
TonB3/TolA      -----------YKGWRGVFYHKASVSALIMITKDGEFDYTILSYSDFKDYNKSVMTLLDD
                           *         ..   . : :  :*..    :  ..  .  .       .

TonB1           IAYKDYPRPKTKTLIRIRVRYYLGGN-
TonB2           SAAHLFPKPEETVHLKIPIAYSLKED-
TonB3/TolA      LKKVDFPPYPGGNMISIKVNFTTKEEQ
                     :*       : * : :    :
```

Supplemental Figure 2

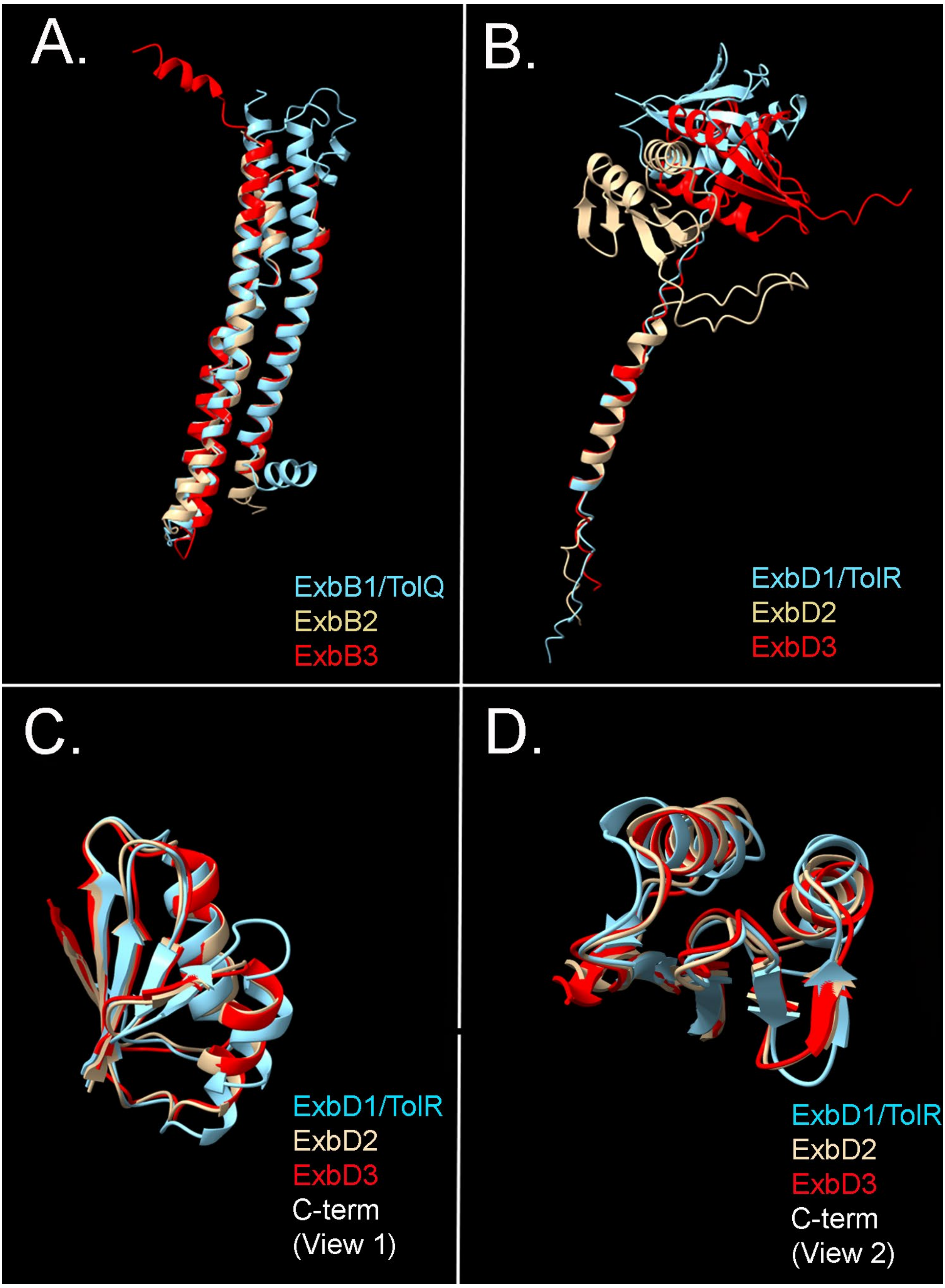

Supplemental Figure 3

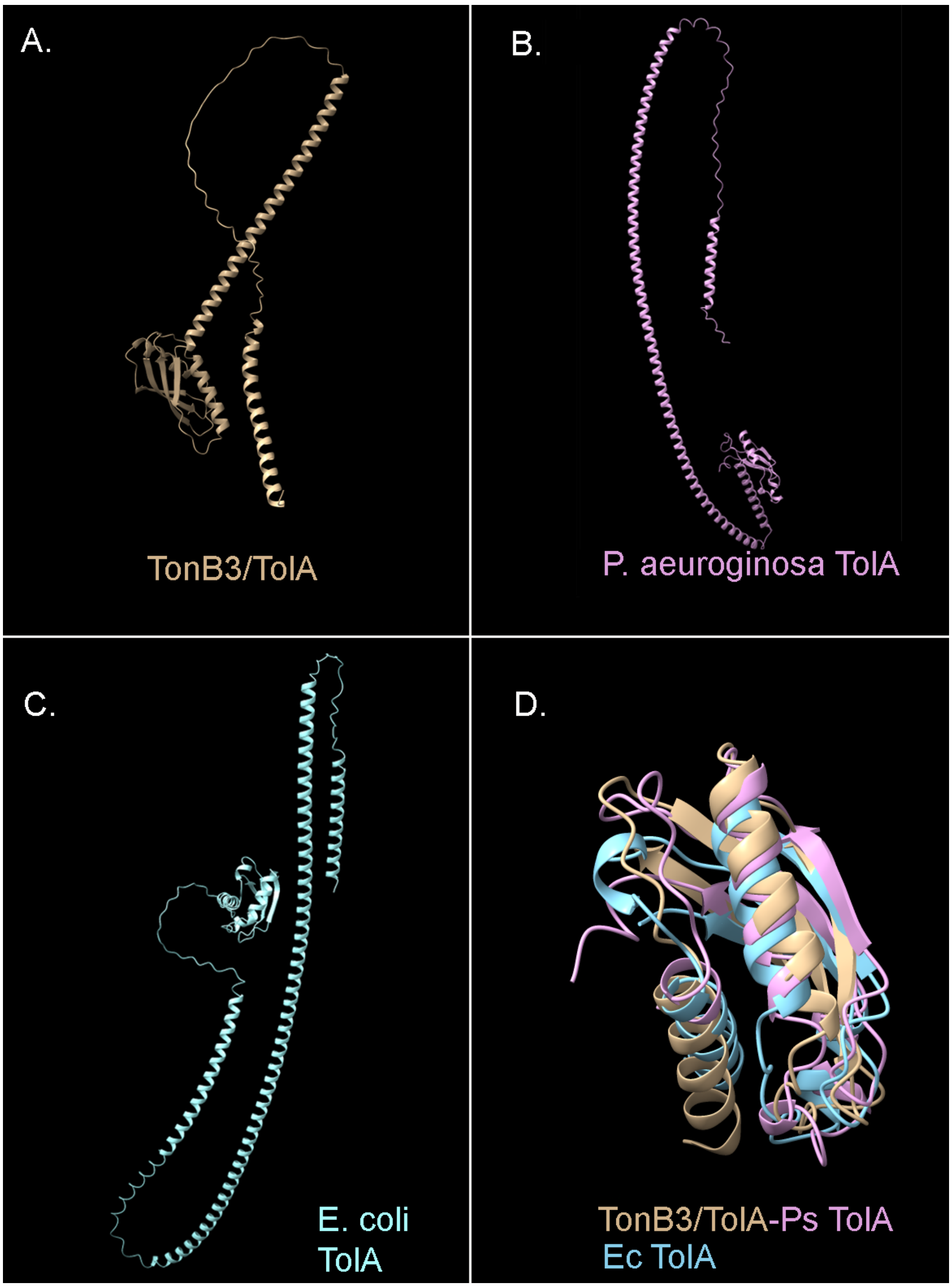

Supplemental Figure 4

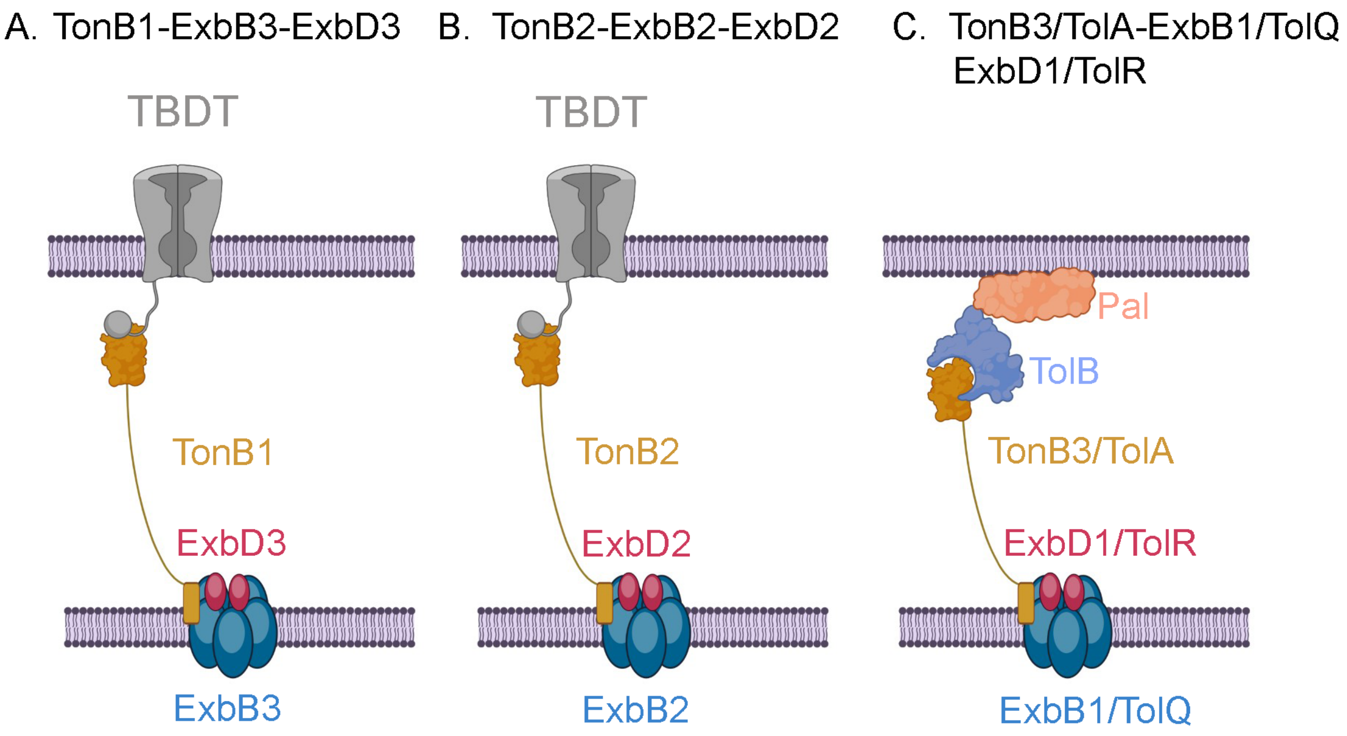

Supplemental Figure 5.

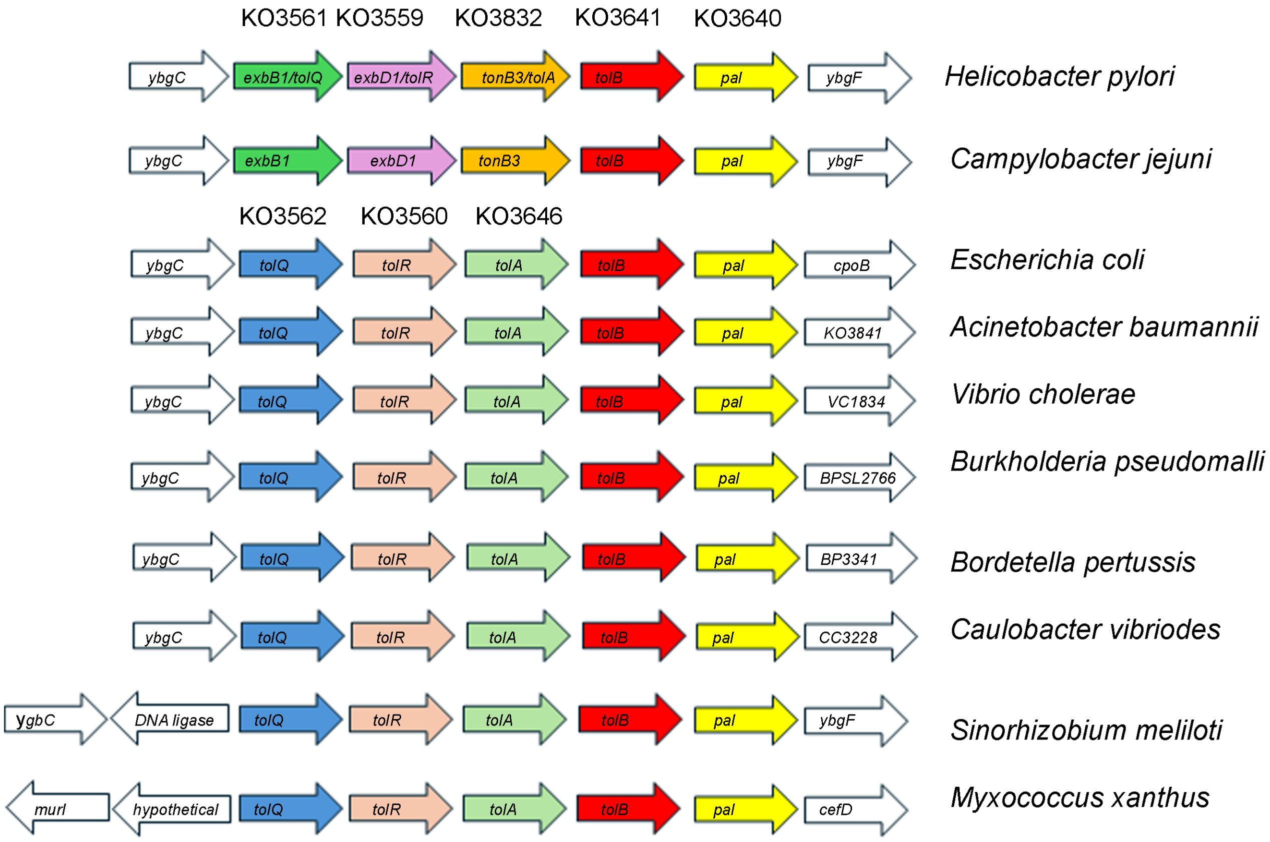

Supplemental Figure 6.

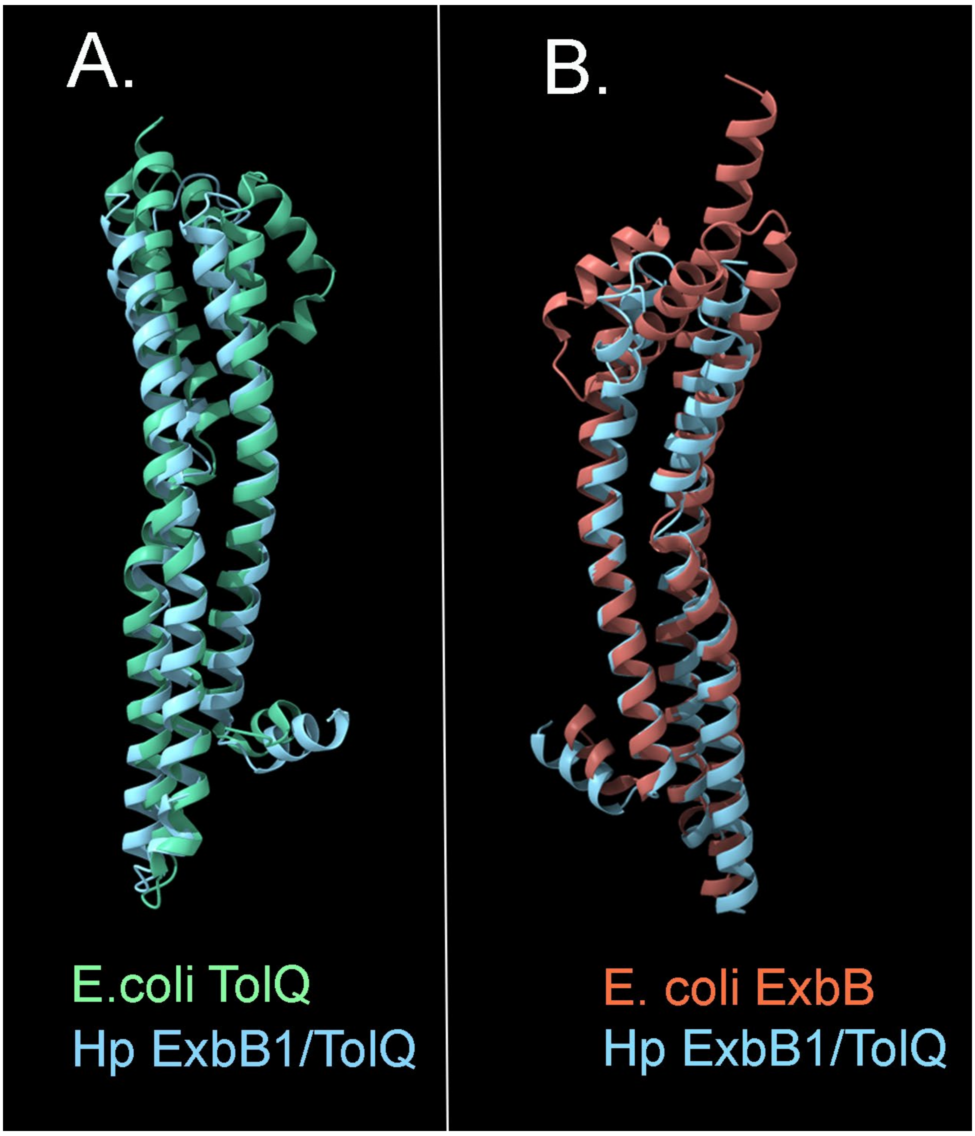

Supplemental Figure 7.

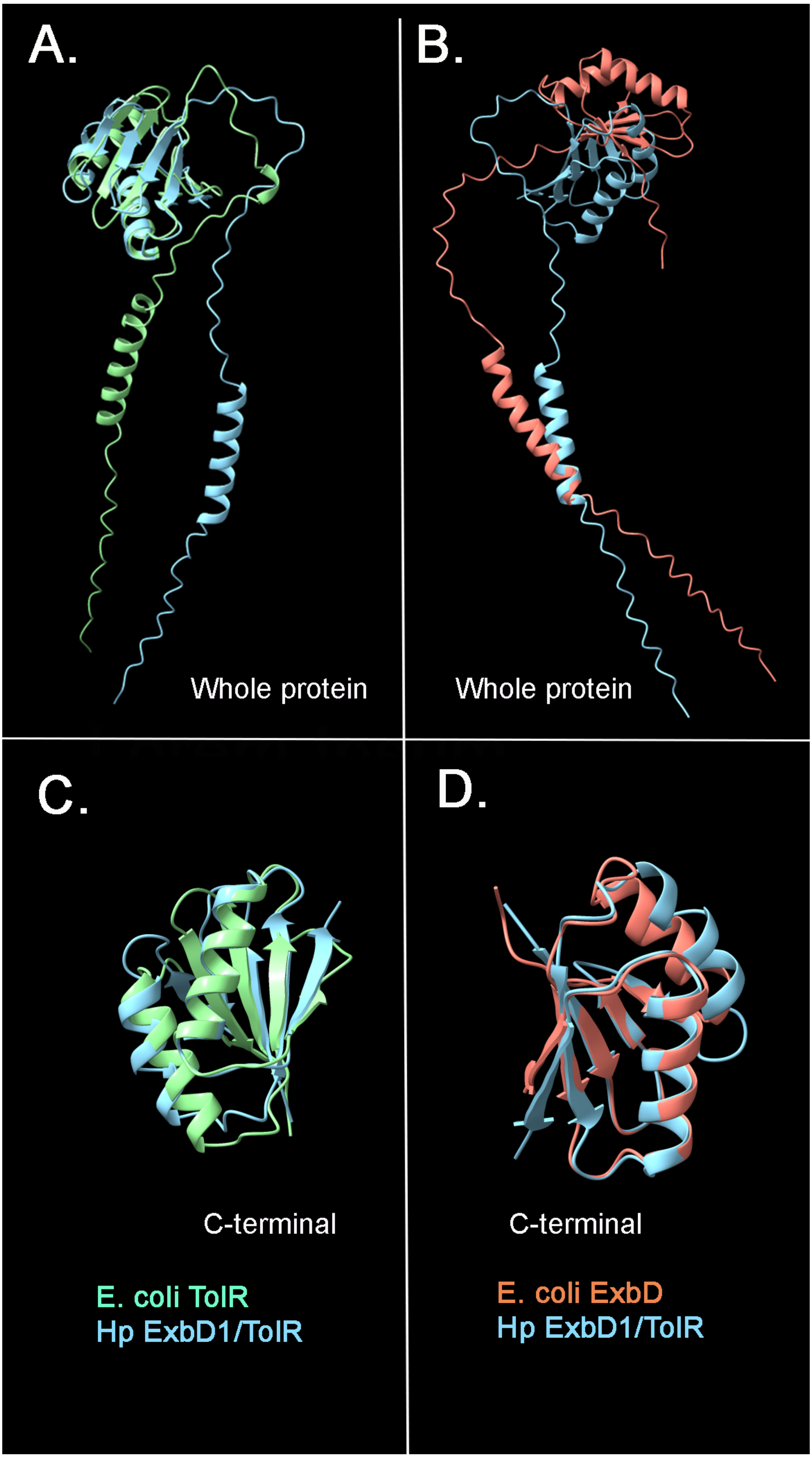

Supplemental Figure 8.
