# Supplementary material for: Multiple TonB-dependent transport systems in Helicobacter pylori
Source: Infect Immun. 2026 Jun 4;94(7):e00018-26. doi: 10.1128/iai.00018-26 (PMC13367063; doi:10.1128/iai.00018-26)
Supplement: Supplemental material — Supplemental figure legends. [file iai.00018-26-s0002.pdf]

Supplemental Figure Legends

Supplemental Figure S1. Sequence relatedness of *H. pylori* TonB1 and TonB2. ClustalW analysis of TonB1 and TonB2 (from *H. pylori* strain 26695) reveals only 29% amino acid identity between the two proteins. Regions rich in proline, lysine and glutamate residues are highlighted in boxes.

Supplemental Figure S2. Structural features of *H. pylori* TonB3/TolA. **A, B:** AlphaFold 3 modeled structures of TonB3/TolA from *H. pylori* strain B8 (1) (panel A) and TonB3/TolA-HA Tag (panel B). *H. pylori* strain B8 is closely related to strain 7.13 (2). An HA Tag inserted into the loop region is shown graphically in red. **C,** ClustalW analysis of *H. pylori* TonB1 (strain 26695), TonB2 (strain 26695) and TonB3/TolA (strain B8 selected for analysis because TonB3/TolA in *H. pylori* strain 26695 contains a split ORF). The boxed sequences indicate the proline-rich regions in TonB1, TonB2 and TonB3/TolA. In comparison to TonB1 and TonB2, TonB3/TolA contains a shorter proline-rich region.

Supplemental Figure S3. Structural similarity among *H. pylori* ExbB and ExbD proteins. **A,** Alignment of AlphaFold 3 modeled structures of ExbB1/TolQ, ExbB2, and ExbB3 (entire proteins) from *H. pylori* strain 26695 are shown (UCSF ChimeraX 1.10.1). RMSD was calculated using UCSF ChimeraX 1.10.1, and RMSD values are reported either across all atom pairs or pruned by ChimeraX to discard outlier atom pairs (cutoff >2 Å). This analysis yielded a RMSD of 0.75Å between ExbB1/TolQ and ExbB2 across 79 pruned atom pairs (7.93 Å across all 145 pairs). An RMSD of 0.68Å was calculated between ExbB2 and ExbB3 across 127 pruned atom pairs (1.11 Å across 137 pairs). **B,** Alignment of AlphaFold 3 predicted structures (entire protein) of ExbD1/TolR, ExbD2, ExbD3. RMSD values were 25.95 Å (entire protein) and 0.26 Å between 19 pruned atom pairs of ExbD1 and ExbD2. RMSD values were 23.87 Å (entire protein) and 0.43 Å between 28 pruned atom pairs of ExbD2 and ExbD3. RMSD values for comparisons of the full-length proteins are high, likely due to several predicted flexible or low-confidence regions (i.e. 70>pLDDT>50) in ExbD1/TolR, ExbD2, and ExbD3. **C, D:** Alignment of AlphaFold 3-predicted structures (two different views) of the C-terminal end (predicted with high confidence, i.e. pLDDT>70) of ExbD homologs [ExbD1/TolR (aa 59-128), ExbD2 (aa 58-125), ExbD3 (aa 58-125)]. The RMSD values for comparisons of C-terminal ExbD2 and C-terminal ExbD1/TolR were 1.71 Å across all 66 pairs, and 0.72 Å across all 67 pairs for C-terminal ExbD2 and C-terminal ExbD3.

Supplemental Figure S4. Structural comparisons of *H. pylori* TolB3/TolA with TolA proteins from other bacterial species. Predicted structures (entire protein) for **(A)** *H. pylori* TonB3/TolA (uniprot D7FCM3), based on *H. pylori* strain B8; **(B)** *P. aeruginosa* TolA (uniprot P50600); and **(C)** *E. coli* TolA (uniprot P19934), based on AlphaFold 3 modeling. **Panel D**

compares the AlphaFold 3 predicted C-terminal regions of *H. pylori* TonB3/TolA (aa159-263) (pLDDT>70), *P. aeruginosa* TolA (aa 286-366, pLDDT>90) and *E. coli* TolA (aa 335-421, pLDDT>90, AlphaFold 3). The RMSD for TonB3/TolA and *E. coli* TolA was 1.44 Å (26 pruned atom pairs) and 5.06 Å across all 77 pairs. The RMSD was 1.22 Å (27 pruned atom pairs) and 1.19 Å (across all 70 pairs) between TonB3/TolA and *P. aeruginosa* TolA. RMSD was calculated using UCSF ChimeraX 1.10.1, and RMSD values are reported either across all atom pairs or pruned by ChimeraX to discard outlier atom pairs (cutoff >2 Å).

Supplemental Figure S5. Schematic showing proteins that comprise TonB1, TonB2 and TonB3/TolA complexes. **A,** Immunopurification of TonB1-HA in strain JL1031 (Table 2) resulted in the copurification of ExbB3 and ExbD3 (Table 3). **B,** Immunopurification of TonB2-HA in strain JL1028 (Table 2) resulted in the copurification of ExbB2 and ExbD2 (Table 3). **C,** Immunoprecipitation of TonB3/TolA-HA in strain JL1033 (Table 2) resulted in the copurification of ExbB1/TolQ and ExbD1/TolR (Table 3). TonB3/TolA-HA immunopurification also yielded TolB, a member of the Tol-Pal system (Table 3). This latter result suggests that TonB3/TolA may be a member of the Tol-Pal system (3-5). ExbB/ExbD subcomplexes in *E. coli* have been reported to have varying oligomeric states (5/1, 6/3 or 5/2 ratios of ExbB/ExbD proteins) (6-9). A 5/2 ratio of TolQ/TolR has been reported in *E. coli* (7, 10).

Supplemental Figure S6. Genome organization of genes encoding components of the Tol system in representative Gram-negative bacterial species. The Tol-Pal system comprises *tolQ*, *tolR*, *tolA*, *tolB* and *pal* genes. The organization of these genes is conserved in most Gram-negative bacteria. In *H. pylori* strain B8 and *Campylobacter jejuni*, *tolQ*, *tolR*, *tolA* have been annotated as *exbB*, *exbD*, and *tonB3,* respectively. In the current study, we designate these genes as *exbB1/tolQ, exbD1/tolR* and *tonB3/tolA* respectively. The KEGG Orthology (KO) numbers, representing molecular functions of functional orthologs, are shown above the relevant genes. The associated KEGG orthology numbers are shown for *H. pylori* ExbB1 (KO3561), *H. pylori* ExbD1 (KO3559), *H. pylori* TonB3 (KO3832), TolB from many species (KO3641), and Pal from many species (KO3640). The KEGG orthology numbers for TolQ (KO3562), TolR (KO3560), and TolA (KO3646) are relevant for many non-*H. pylori* species.

Supplemental Figure S7. Predicted structure of *H. pylori B8* ExbB1/TolQ (HPB8_371) is similar to that of *E. coli* TolQ and *E. coli* ExbB. The predicted structure of *H. pylori B8* ExbB1/TolQ is based on AlphaFold 3 analysis, while the structures of *E. coli* TolQ [pdb_9ddm (7)] and *E. coli* ExbB [pdb_5zfp (9)] were experimentally determined. The RMSD values for comparisons of *H. pylori* ExbB1/TolQ and *E. coli* TolQ were 1.09 Å across 106 pruned atom pairs and 6.14 Å across all 187 pairs **(panel A)**. The RMSD values for

comparisons of ExbB1/TolQ and *E. coli* ExbB were 1.07 Å between 95 pruned atom pairs and 5.53 Å across all 187 pairs **(panel B)**.

Supplemental Figure S8. Predicted structure of *H. pylori B8* ExbD1/TolR (HPB8_372) is similar to that of *E. coli* TolR. Comparison of AlphaFold 3-predicted structures of *H. pylori* B8 ExbD1/TolR with AlphaFold 3-modeled structures of *E. coli* TolR (Uniprot P0ABV6, **panel A)** and *E. coli* ExbD (uniprot P0ABV2, **panel B**). The RMSD between 37 pruned atoms pairs for *H. pylori* B8 ExbD1/TolR with *E. coli TolR* (panel A) is 0.997Å (12.32Å across all 133 pairs). The RMSD between 49 pruned atom pairs for *H. pylori* B8 ExbD1/TolR with *E. coli* ExbD (panel B) is 1.30 Å for 9 pruned atom pairs (across all 130 pairs: 17.15 Å). The high level of flexibility likely contributed to the high RMSD values reported for entire protein comparisons. **C, D,** Comparison of AlphaFold 3-predicted structures (pLDDT>70) of the C-terminal end of *H. pylori* ExbD1/TolR (aa 59-128) with *E. coli* TolR (aa 61-138) (panel C), and *E. coli* ExbD (aa 58-132) (panel D). The RMSD values for comparisons of C-terminal end of *H. pylori* ExbD1/TolR with *E. coli* TolR was 0.944 Å between 29 pruned atoms (4.18 Å across all 76 pairs) and the RMSD for comparison of C-terminal ExbD1/TolR with *E. coli* ExbD was 0.72 Å (2.12 Å across all 67 pairs).

1. Farnbacher M, Jahns T, Willrodt D, Daniel R, Haas R, Goesmann A, Kurtz S, Rieder G. 2010. Sequencing, annotation, and comparative genome analysis of the gerbil-adapted *Helicobacter pylori* strain B8. BMC Genomics 11:335.
2. Franco AT, Israel DA, Washington MK, Krishna U, Fox JG, Rogers AB, Neish AS, Collier-Hyams L, Perez-Perez GI, Hatakeyama M, Whitehead R, Gaus K, O'Brien DP, Romero-Gallo J, Peek RM, Jr. 2005. Activation of beta-catenin by carcinogenic *Helicobacter pylori*. Proc Natl Acad Sci U S A 102:10646–51.
3. Szczepaniak J, Press C, Kleanthous C. 2020. The multifarious roles of Tol-Pal in Gram-negative bacteria. FEMS Microbiol Rev 44:490–506.
4. Cascales E, Gavioli M, Sturgis JN, Lloubes R. 2000. Proton motive force drives the interaction of the inner membrane TolA and outer membrane pal proteins in *Escherichia coli*. Mol Microbiol 38:904–15.
5. Bradbeer C. 1993. The proton motive force drives the outer membrane transport of cobalamin in *Escherichia coli*. J Bacteriol 175:3146–50.
6. Celia H, Botos I, Ni X, Fox T, De Val N, Lloubes R, Jiang J, Buchanan SK. 2019. Cryo-EM structure of the bacterial Ton motor subcomplex ExbB-ExbD provides information on structure and stoichiometry. Commun Biol 2:358.

7. Celia H, Botos I, Ghirlando R, Duche D, Beach BM, Lloubes R, Buchanan SK. 2025. Cryo-EM structures of the *E. coli* Ton and Tol motor complexes. Nat Commun 16:5506.
8. Celia H, Noinaj N, Zakharov SD, Bordignon E, Botos I, Santamaria M, Barnard TJ, Cramer WA, Lloubes R, Buchanan SK. 2016. Structural insight into the role of the Ton complex in energy transduction. Nature 538:60–65.
9. Maki-Yonekura S, Matsuoka R, Yamashita Y, Shimizu H, Tanaka M, Iwabuki F, Yonekura K. 2018. Hexameric and pentameric complexes of the ExbBD energizer in the Ton system. Elife 7.
10. Williams-Jones DP, Webby MN, Press CE, Gradon JM, Armstrong SR, Szczepaniak J, Kleanthous C. 2023. Tunable force transduction through the *Escherichia coli* cell envelope. Proc Natl Acad Sci U S A 120:e2306707120.
