# Supplementary material for: Multiple TonB-dependent transport systems in Helicobacter pylori
Source: Infect Immun. 2026 Jun 4;94(7):e00018-26. doi: 10.1128/iai.00018-26 (PMC13367063; doi:10.1128/iai.00018-26)
Supplement: Table S1 — LC-MS/MS analysis of proteins immunopurified from H. pylori strains harboring ExbD2-FLAG, LolF-FLAG, FrdC-FLAG. [file iai.00018-26-s0003.pdf]

Supplemental Table 1. LC-MS/MS analysis of proteins immunopurified from H. pylori strains harboring ExbD2-FLAG, LolF-FLAG, FrdC-FLAG[a]															
Immunopurified Protein (Gene Number) [b,c]	Bait protein														
	ExbD2-FLAG[d,e]					LolF-FLAG[d,e]						FrdC-FLAG[d,e]			
	Exp1	Exp2	Exp3	Exp4		Exp1	Exp2	Exp3	Exp4	Exp5		Exp1	Exp2	Exp3	Exp4
ExbD2 (HP1340)	274	282	128	151		0	1	3	2	2		21	42	19	5
ExbB2 (HP1339)	375	551	224	244		0	9	7	0	2		37	70	15	5
TonB2 (HP1341)	70	135	14	17		0	6	8	5	7		1	3	4	2
TonB1 (HP0582)	0	0	0	0		0	0	0	0	0		0	0	0	0
(ExbB1/TolQ) (HP1130)	0	1	0	2		0	2	3	3	2		1	1	0	0
ExbD1/TolR (HP1129)	0	0	0	0		0	0	0	0	0		0	0	0	0
TonB3/TolA (HP1128/ HP1127)	0	0	0	0		0	0	0	0	0		0	0	0	0
ExbB3 (HP1445)	16	29	11	14		0	2	2	0	1		0	0	0	2
ExbD3 (HP1446)	9	28	2	5		0	2	7	0	0		3	8	1	0
LolF (HP0787)	1	8	2	0		549	804	665	316	372		0	22	4	2
FrdC (HP0193)	0	7	1	1		0	14	11	3	3		431	435	214	201

[a]Results presented are extracted from a previous study (1) involving LolF-FLAG proteins. ExbD2-FLAG and FrdC-FLAG were used as control pull downs in the study. Relevant data pertaining to TonB related target proteins (i.e. ExbB, ExbD, TonB) as well as LolF and FrdC are shown in the current supplemental table.

[b]TonB related proteins and gene numbers in *H. pylori* strain 26695 identified by MS. Included in the Table are protein levels for control pull downs (LolF, FrdC)

[c]Immunoprecipitation was performed with *H. pylori* strains harboring ExbD2-FLAG, LolF-FLAG, FrdC-FLAG (1).

[d]Spectral count data presented for affinity purification were normalized based on the mean of total spectral counts for each experiment

[d] 4, 5 and 4 independent experiments were conducted with ExbD2-FLAG, LolF-FLAG and FrdC-FLAG strains, respectively.

1. McClain MS, Bryant KN, McDonald WH, Algood HMS, Cover TL. 2023. Identification of an Essential LolD-Like Protein in *Helicobacter pylori*. J Bacteriol 205:e0005223.
